# Evidence of Organ-Specific Metal Accumulation: ICP-MS Elemental Analysis of Autopsy Tissues of Tobacco Smokers

**DOI:** 10.3390/ijms26136368

**Published:** 2025-07-02

**Authors:** Wojciech Flieger, Przemysław Niedzielski, Zofia Wojciechowska, Aleksandra Proch, Jędrzej Proch, Alicja Forma, Andrzej Torbicz, Dariusz Majerek, Grzegorz Teresiński, Jacek Baj, Ryszard Maciejewski, Jolanta Flieger

**Affiliations:** 1 Department of Plastic Surgery, St. John’s Cancer Center, Jaczewskiego 7, 20-090 Lublin, Poland; 2 Institute of Health Sciences, John Paul II Catholic University of Lublin, Konstantynów 1 H, 20-708 Lublin, Poland; ryszard.maciejewski@kul.pl; 3 Doctoral School, Medical University of Lublin, Aleje Racławickie 1, 20-059 Lublin, Poland; 4 Department of Analytical Chemistry, Faculty of Chemistry, Adam Mickiewicz University, ul. Uniwersytetu Poznańskiego 8, 61-614 Poznań, Poland; przemyslaw.niedzielski@amu.edu.pl (P.N.); zofia.wojciechowska@amu.edu.pl (Z.W.);; 5 Department of Forensic Medicine, Medical University of Lublin, Jaczewskiego 8b, 20-090 Lublin, Poland; aforma@onet.pl (A.F.); grzegorz.teresinski@umlub.pl (G.T.); 6 Department of Analytical Chemistry, Medical University of Lublin, Chodźki 4a (Collegium Pharmaceuticum), 20-093 Lublin, Poland; andrzej.torbicz@umlub.pl; 7 Department of Applied Mathematics, Lublin University of Technology, Nadbystrzycka 38, 20-618 Lublin, Poland; d.majerek@pollub.pl; 8 Department of Correct, Clinical and Imaging Anatomy, Medical University of Lublin, ul. Jaczewskiego 4, 20-090 Lublin, Poland; jacek.baj@umlub.pl

**Keywords:** human tissues, toxic metals, elemental homeostasis, bioaccumulation, multi-elemental analysis, tobacco smokers

## Abstract

Cigarette smoking exposes individuals to numerous toxic substances, including heavy metals. Smokers are at risk due to the accumulation of these substances in various tissues. Objective: To compare the concentrations of 41 elements in 11 brain regions, the spinal cord, the bronchial, the lungs, and the liver in smokers (*n* = 11) and non-smokers (*n* = 17). Elemental composition was determined by ICP-MS after wet digestion in a microwave system. The following toxic elements were detected at levels of µg/g w.w.: Al, Cd, Pb, Ba, As, Ni, and Tl. Significantly higher concentrations of Al were detected in bronchial and lung, and more Pb, Tl, and rare earth elements were detected in the liver of smokers compared to non-smokers. In addition, smokers had significantly lower concentrations of essential elements involved in antioxidant defense, such as Cu, in liver tissue (*p* = 0.033). The brain and spinal cord in smokers and non-smokers were similar in terms of chemical composition, except the insula, where smokers had greater Al accumulation (*p* = 0.030), the precentral gyrus, where higher amounts of As, Cd, and Mn were detected, and the septal nucleus accumbens, which preferentially accumulated Cd in smokers; however, the *p*-values indicate that these differences were not statistically significant. Most brain areas of smokers were characterized by higher Na content (*p* < 0.05). These findings prove the long-term effects of smoking, demonstrating the bioaccumulation of toxic elements, the increased levels of rare earth elements in the liver, decreased levels of elements involved in the body’s antioxidant defense, and disruption of sodium homeostasis in the brain of smokers.

## 1. Introduction

Nicotine addiction and tobacco smoking are the leading causes of premature death [1,2]. According to the World Health Organization (WHO), one person dies every 4 s as a result of tobacco smoking [3]. In Poland, according to the National Health Fund’s report on tobacco-related diseases in 2021, 8.5 million people smoked tobacco. Tobacco smoking is the most common identified cause of chronic obstructive pulmonary disease (COPD) and coexisting diseases, such as chronic bronchitis, emphysema, and recurrent bacterial lung infections [4]. The WHO currently predicts that COPD will become the third leading cause of death worldwide by 2030 [5]. Tobacco-related diseases also include malignancies, including lung, laryngeal, and oropharyngeal cancers, and cardiovascular diseases, including ischemic heart disease [6,7]. Recent population studies have confirmed in animal models that chronic exposure to tobacco smoke accelerates aging through mitochondrial dysfunction [8]. Unfortunately, the use of tobacco products and the prevalence of cigarette smoking continue despite evidence of the harmful effects of smoking [9,10].

Tobacco use is declining worldwide, but it is still a major public health problem. WHO data shows that in 2022, about 1.25 billion adults consumed tobacco, down from 32.7% of the world’s population in 2000. However, the use of alternative smoking products (e.g., electronic cigarettes, heated tobacco products (HPT)) is growing, especially among young people. Among the observed trends, the attitude of minors to tobacco is worrying. According to the report, about one in ten adolescents aged 13 to 15 (9.7% or about 37 million) consume tobacco products. In Poland, according to the 2022 Global Youth Tobacco Survey (GYTS): 11.7% of Polish youth aged 13–15 smoke traditional cigarettes; 22.3% of Polish youth aged 13–15 use electronic cigarettes [11]. Moreover, although smoking prevalence is decreasing, polyconsumption (the combined use of different smoking products) is increasing, especially among young people. In Italy, for example, 30.2% of young people use at least one product from traditional cigarettes, heated tobacco or electronic cigarettes. Although cigarette prevalence among 13–15-year-olds in Italy decreased from 21% in 2010 to 15% in 2022, the prevalence of e-cigarette use increased from 8% in 2014 to 20% in 2022 [12].

The prevalence of cigarette smoking has even been studied among university athletes at Thammasat University in Thailand, with a mean age of 19.8 ± 1.3 years [13]. The study confirmed that the proportion of smokers in the population studied was relatively high. Smokers were predominantly men (70.6% vs. 29.4%, *p* < 0.001) who had higher levels of the measured indicator, exhaled carbon monoxide gas (CO; 3.75 ± 3.08 ppm vs. 2.18 ± 0.73 ppm, *p* < 0.001). Cigarette smoke has been shown to contain many toxic substances, including volatile organic compounds, nitrosamines, polycyclic aromatic hydrocarbons, and toxic heavy metals [14,15,16]. Some chemical elements penetrate tobacco smoke better than others, with concentrations ranging from almost 1% (As compounds) to up to 22% (Cd compounds) [17,18,19]. Edgar Pinto et al. [18] showed that the transfer of Tl and Cd to cigarette smoke was >81%; As and Pb were transferred at rates of 33–60%.

Several reports describe the determination of heavy metals in different cigarette brands [20,21,22,23]. A 2024 study by Tarimo Felix et al. reported the content of heavy metals (As, Cd, Cr, Hg, and Pb) in different brands of cigarettes produced worldwide [24]. Fresquez et al. investigated the content of As, Be, Cd, Cr, Co, Pb, Mn, Hg, and Ni in 50 commercial tobacco products available in the USA using inductively coupled plasma mass spectrometry (ICP MS) [25]. The mean values of Mn ranged from 131 to 245 μg/g, Cr values ranged from 1.4 to 3.2 μg/g, As values ranged from 0.22 to 0.36 μg/g, Cd values ranged from 1.0 to 1.7 μg/g, Ni values ranged from 2.1 to 3.9 μg/g, and Pb values ranged from 0.6 to 1.2 μg/g. In the study by Kazi et al., toxic metals (Al, Cd, Ni, and Pb) were detected in the ash of different brands of cigarettes available in Pakistan, with Al being the most abundant [26]. As reported by the International Agency for Research on Cancer, these metals are carcinogenic [27]. In this context, in March 2012, the US Food and Drug Administration (FDA) published guidelines for reporting harmful ingredients in tobacco products, including As, Cd, Cr, Ni, and Pb [28]. Matassa et al. [29] provided direct evidence, using microscopic imaging, that metallic inorganic microparticles are transported with the gaseous smoke stream to the fibrous filters in the mouthpiece and form nanostructures of up to 150 µm in size containing, among others, vanadium, chromium, iron, nickel, copper, uranium, manganese, and osmium. The authors point out that the insufficient porosity of the filters is not an obstacle for toxic volatile substances. The studies provide a basis for further work developing microfibers that are capable of capturing toxic particles in gaseous media to protect human health.

The presence of metals in tobacco products results from the ability of tobacco (*Nicotiana tabacum* L.) to accumulate heavy metals during plant growth [30]. The tobacco absorbs metal ions and compounds from the soil through its roots, which are then transported to the leaves [31,32]. The efficiency of the uptake of contaminants from the soil depends on plant growth conditions, soil pH and type, organic matter composition, presence of organic or metal ions, type of fertilizer used, and geographical location [17,33]. Therefore, the origin of the tobacco may play a key role in the level of heavy metal contamination in the variety of tobacco brands [34,35,36,37,38]. The source of soil contamination with heavy metals such as arsenic (As), cadmium (Cd), chromium (Cr), mercury (Hg), and lead (Pb) is mainly anthropogenic human activity (wastewater, spraying, fertilizers, mining, and atmospheric deposition) [39]. It has been shown that Cd accumulates in the tobacco plant in a natural way [30]. This “hyperaccumulation” leads to very high cadmium concentrations in tobacco leaves, which are relatively independent of soil content [30,40]. The cadmium content of tobacco leaves is in the range of 1 to 2 µg/g of dry weight, which leads to amounts of 0.5 to 1 µg of cadmium per cigarette. Another possible source of contamination may be the cigarette manufacturing process itself and additives in the form of flavorings or humectants [41].

Tobacco smoke is a source of exposure to toxic elements [42]. Metals found in cigarette smoke are carcinogenic, toxic to the cardiovascular system and kidneys (As and Cd), and toxic to the nervous system (Pb) [43]. Chronic smoking leads to the accumulation of metals in tissues and body fluids, resulting in the deterioration of health [44]. Pinto et al. [20] demonstrated accumulation in the lungs of smokers (As, Cd, and Pb). Biomonitoring studies have shown that active and passive smokers have significantly elevated levels of Cd and Pb [45,46]. These elements have a long half-life, i.e., 10–12 years [47]. Stojanović et al. [48] showed elevated levels of nickel in the blood and urine of smokers. Richter et al. [49] showed that smokers had significantly higher urinary levels of Cd, Pb, Sb, and Ba compared to non-smokers. The exposure of children of about 7–8 years of age to tobacco smoke is one of the sources of increased exposure to heavy metals. In addition to cotinine (a metabolite of nicotine), traces of chromium (Cr), copper (Cu), lead (Pb), manganese (Mn), nickel (Ni), and zinc (Zn) were found in the saliva of exposed children [50]. The authors observed significant associations between cotinine levels and salivary levels of Cu, Zn, and Pb [50]. However, most studies of tobacco smoke poisoning focus on cadmium, which has been considered a toxin associated with lung disease since 1950 [51,52,53,54,55]. The National Health and Nutrition Examination Survey (NHANES) has been continuously monitoring urinary cadmium concentrations since 1988 to assess trends in cadmium exposure, including those from cigarette smoking.

However, knowledge of local cadmium deposition, e.g., in the lungs, is limited [53]. It is thought that the uptake of cadmium oxide produced during smoking into the lungs is much higher than the uptake of cadmium from food through the gut, and therefore, the blood and urine cadmium concentrations of smokers may be several times higher than those of non-smokers [53,56,57,58,59,60,61]. The local accumulation of cadmium in the lungs appears to be a critical component of the predisposition to lung disease in long-term smokers. This is particularly important given that the biological half-life of cadmium in the human body is >25 years, which is a significant period, suggesting that cadmium may be significantly retained in the lungs of long-term smokers [53]. At this stage of research, it is difficult to disentangle the effects of the different components of tobacco smoke. The presence of toxic metals in body fluids suggests that accumulation in tissues, lungs, and other organs is likely. Such accumulation may affect intracellular signaling and, consequently, host defense functions. This explains the increased susceptibility to bacterial colonization and infection, leading to chronic inflammation, fibrosis, and emphysema, and further impairment of lung ventilatory function and gas exchange, key clinical features of severe COPD observed in smokers. Further studies are needed to investigate the relationship between tobacco smoke exposure and pathogenic mechanisms, and to determine the metal concentrations in different sites in long-term smokers. Such studies can significantly improve the understanding of the pathogenic significance of toxic element accumulation due to tobacco smoking and the associated potential to use this knowledge in risk monitoring.

This study shows, for the first time, the levels of many chemical elements in post-mortem tissues from 11 different areas of the brain, spinal cord, lung, bronchus, and liver of chronic smokers compared to a control group of non-smokers. The aim is to understand the long-term effects of smoking on disturbances in the levels of essential elements, as well as the organ-specific accumulation of toxic elements. The study provides up-to-date evidence for the need to monitor tobacco products for certain elements that tend to accumulate in tissues.

## 2. Results

### 2.1. Evidence of Metal Accumulation in Bronchial Tissues of Smokers

The bronchial tissues of smokers/non-smokers were analyzed for the content of 41 metals and metalloids using ICP-MS. The descriptive statistics developed based on the measured data are summarized in Table S1a, Supplementary Materials. The elemental composition of bronchial tissues revealed a high concentration range 100–2000 µg/g (wet weight, w.w.) for essential macroelements, including sodium (Na), potassium (K), phosphorus (P), calcium (Ca), and magnesium (Mg). Trace elements, such as iron (Fe), zinc (Zn), rubidium (Rb), strontium (Sr), copper (Cu), manganese (Mn), selenium (Se), cadmium (Cd), and lead (Pb), were detected within the range of 20–0.04 µg/g (w.w.). Additionally, ultra-trace elements, namely chromium (Cr), vanadium (V), cobalt (Co), nickel (Ni), and cerium (Ce), were present at concentrations between 0.01 and 0.002 µg/g (w.w.). The distribution of these elements suggests both physiological relevance and potential environmental exposure influences.

**Figure 1:**
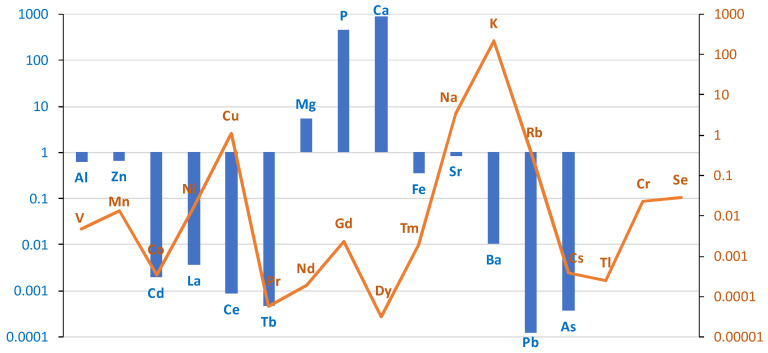
The difference between the mean values of metal concentrations (µg/g w.w.) of bronchial tissue collected from the study and control groups. Blue bar represents log(case mean–control mean); orange line represents log(control mean–case mean).

The comparison of the case/control groups is presented as a graph (Figure 1), with the difference between the mean values of the chemical element concentrations presented in µg/g (w.w.) of tissue collected from the study and control groups. Bronchial tissues from smokers showed a higher content of Al, Zn, Cd, La, Ce, Tb, Mg, P, Ca, Fe, Sr, Ba, Pb, and As compared to the tissue of non-smokers. The remaining elements were found in higher concentrations in the bronchial tissue of the non-smokers group: V, Mn, Co, Ni, Cu, Pr, Nd, Gd, Dy, Tm, Na, K, Rb, Cs, Tl, Cr, and Se. The highest difference was observed for Al, i.e., 0.63 µg/g (w.w.).

A comparison of the content of individual elements in bronchial tissue in smokers and non-smokers is presented in Table 1.

**Figure 2:**
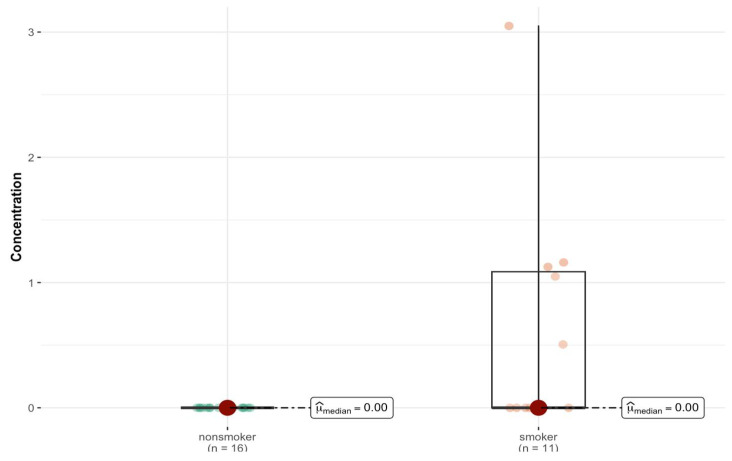
Statistically significant difference in Al concentration (µg/g w.w.) in bronchial tissue of smokers (*n* = 11) compared to non-smokers (*n* = 16).

Based on the results of the Mann–Whitney test, it can be seen that, for most elements, no statistically significant differences in concentration were observed between the smoking and non-smoking groups. The exception is aluminum (Al), for which the *p*-value is 0.004. This indicates a significant difference at the typical level of statistical significance, e.g., 0.05 (Figure 2).

### 2.2. Comparison of Elemental Content in Lung Tissue (Left and Right) Between Smokers and Non-Smokers

Descriptive statistics of elemental content in right and left lung tissue (right and left lung, *n* = 55) for the study population (smokers and non-smokers) are summarized in Supplementary Materials Table S1b,c in the Supplementary Materials. An analysis of lung tissues demonstrated elevated concentrations (1500–50 µg/g; w.w.) of key macroelements, including potassium (K), sodium (Na), phosphorus (P), iron (Fe), calcium (Ca), and magnesium (Mg). Trace elements, such as zinc (Zn), aluminum (Al), rubidium (Rb), copper (Cu), strontium (Sr), manganese (Mn), selenium (Se), cadmium (Cd), chromium (Cr), vanadium (V), and lead (Pb), were found in the range of 10–0.01 µg/g (w.w.). Ultra-trace elements, including cerium (Ce), nickel (Ni), cobalt (Co), cesium (Cs), and neodymium (Nd), were detected at concentrations between 0.01 and 0.001 µg/g (w.w.). The elemental profile reflects both the metabolic requirements of pulmonary tissues and potential accumulation from environmental exposure.

**Figure 3:**
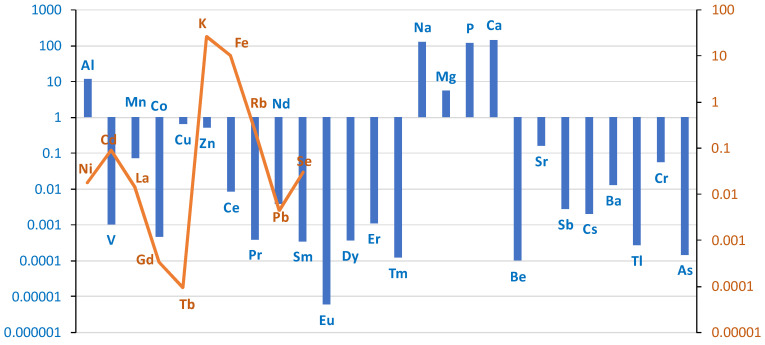
Comparison of differences in mean values of individual elements on a logarithmic scale in lung tissue (right + left) divided into case/control groups. Blue bar represents log(case mean–control mean); orange line represents log(control mean–case mean).

The differences between the groups in the content of individual elements in the lung tissue (right and left) are shown in the logarithmic scale graph in Figure 3. In the case group, higher levels of the following elements were found in the lung tissue: Al, V, Mn, Co, Cu, Zn, Ce, Pr, Nd, Sm, Eu, Dy, Er, Tm, Na, Mg, P, Ca, Be, Sr, Sb, Cs, Ba, Tl, Cr, and As. Lower levels were found for Ni, Cd, La, Gd, Tb, K, Fe, Rb, Pb, and Se.

Higher levels of As and Al were found in the lung tissue of smokers. As levels were in the ng/g range, but were twice as high in smokers as in non-smokers (0.43 ng/g vs. 0.28 ng/g; w.w.). For Al, the mean level was three times higher in smokers than in non-smokers (18 vs. 6.7 µg/g; w.w.). The Ba level was 7.5 times higher in smokers (0.015 vs. 0.002 µg/g). A similar trend was observed in other studies [20], but the values reported by Pinto are much higher due to the conversion of the measurements to dry tissue weight.

**Figure 4:**
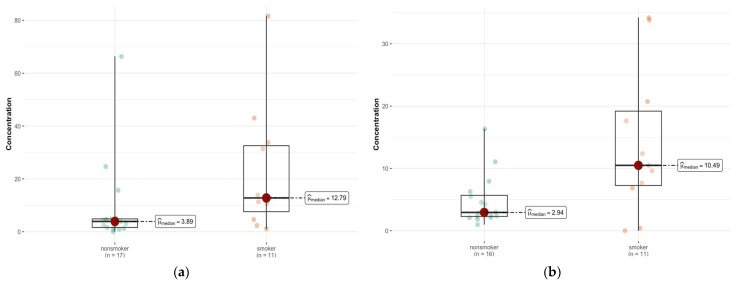
Statistically significant difference in Al concentration (µg/g w.w.) in the left lung (**a**) between smokers (*n* = 11) and non-smokers (*n* = 17), and right lung (**b**) tissue of smokers (*n* = 11) compared with non-smokers (*n* = 16).

When the left and right lung tissues are considered separately, there is an asymmetric distribution of elements between the two sides of the body. A comparative analysis of the medians between the smoker/non-smoker groups (Table 2) revealed statistically significant differences for Al content in both the right (*p* = 0.026) and left lungs (*p* = 0.022) (Figure 4a,b) and for Dy in the right (*p* = 0.035) (Figure S1, Supplementary Materials) and left lungs (*p* = 0.041). Smokers have statistically significantly more of these elements. In the left lung, there were additional differences in the content of Er (*p* = 0.003), Nd (*p* = 0.018), Pr (*p* = 0.004), Sm (*p* = 0.01), Sr (*p* = 0.011), Mn (*p* = 0.047), and Na (*p* = 0.042) (Figure S2, Supplementary Materials).

### 2.3. Comparison of the Content of Elements in Liver Tissue in the Group of Smokers and Non-Smokers

For liver tissue, descriptive statistics are summarized in Table S1d (Supplementary Materials). The elemental analysis of liver tissue showed high concentrations (2000–100 µg/g; w.w.) of major elements, including phosphorus (P), potassium (K), sodium (Na), iron (Fe), and magnesium (Mg). Trace elements, such as calcium (Ca), zinc (Zn), rubidium (Rb), manganese (Mn), copper (Cu), selenium (Se), cadmium (Cd), strontium (Sr), and lead (Pb), were present in the range of 40–0.1 µg/g (w.w.). Ultra-trace elements, including chromium (Cr), cobalt (Co), cesium (Cs), cerium (Ce), and nickel (Ni), were detected at concentrations between 0.02 and 0.002 µg/g (w.w.). This distribution highlights the liver’s central role in metabolism, detoxification, and elemental storage.

**Figure 5:**
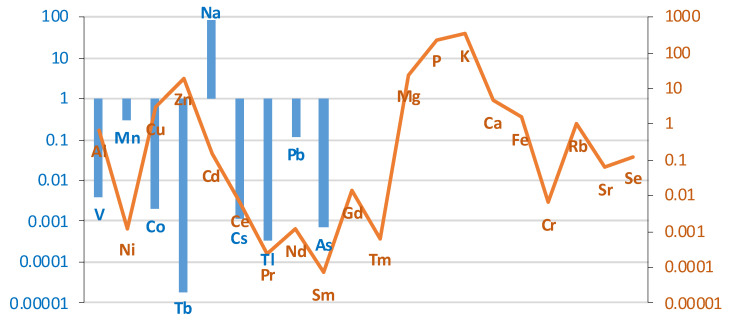
Comparison of the differences between groups (smokers/non-smokers) in the mean levels of individual elements in liver tissue on a logarithmic scale. Blue bar represents log(case mean–control mean); orange line represents log(control mean–case mean).

The differences in the content of individual elements in liver tissue between the group of cases (smokers) and controls (non-smokers) on a logarithmic scale are presented in the graph in Figure 5.

The liver of smokers contained more elements, such as V, Mn, Co, Tb, Na, Cs, Tl, Pb, As, La. The concentration of the remaining elements was lower in the liver of smokers compared to the control group. A comparison of the content of individual elements in liver tissue in smokers and non-smokers is presented in Table 3.

**Figure 6:**
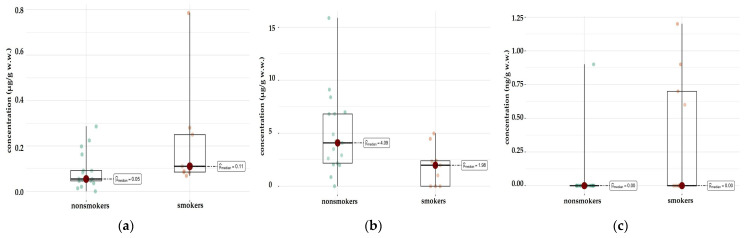
Comparison of statistically significant differences in median values of Pb (**a**), Cu (**b**), and Tl (**c**) content in liver tissue of smokers (*n* = 9) and non-smokers (*n* = 17).

For most elements, the test showed no statistically significant differences. This means that the levels of these elements in the livers of smokers and non-smokers are similar (*p* > 0.05). However, for two elements Cu and Pb, statistically significant differences were observed (Figure 6a,b). For Cu (*p* = 0.033), smokers and non-smokers differ significantly, suggesting that smoking may affect the levels of this element in the liver. There were also significant differences for Pb (*p* = 0.025), which may indicate an increased accumulation of this element in smokers. Also noteworthy is the element Tl (*p* = 0.025), which also shows a statistically significant difference between the groups (Figure 6c).

### 2.4. Comparison of Elemental Content in Different Parts of the Brain Between Smokers and Non-Smokers

**Figure 7:**
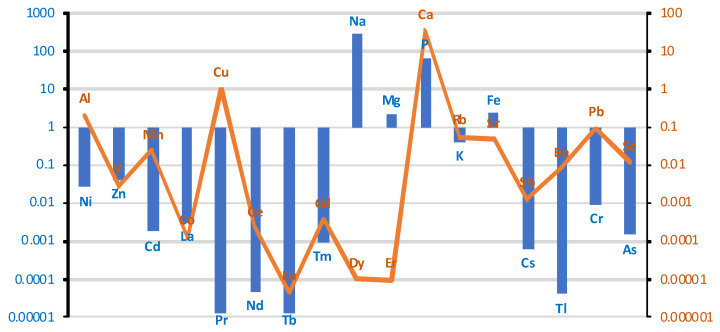
Comparison of differences in mean values of individual elements at a logarithmic scale in brain tissue between smoking/non-smoking groups. Blue bar represents log(case mean–control mean); orange line represents log(control mean–case mean).

Descriptive statistics developed for the study population based on measurements of element levels in brain tissues are summarized in Table S1e (Supplementary Materials). The elemental composition of brain tissues indicated high concentrations (3000–150 µg/g wet weight) of potassium (K), phosphorus (P), sodium (Na), and magnesium (Mg). Trace elements, such as calcium (Ca), iron (Fe), zinc (Zn), copper (Cu), rubidium (Rb), manganese (Mn), aluminum (Al), lead (Pb), selenium (Se), and strontium (Sr), were present in the range of 100–0.1 µg/g (w.w.). Ultra-trace elements, including nickel (Ni), chromium (Cr), cadmium (Cd), barium (Ba), cesium (Cs), and arsenic (As), were detected at concentrations between 0.05 and 0.001 µg/g (w.w.). The elemental profile underscores the brain’s complex biochemical environment and highlights the potential influence of environmental and systemic exposures. Considering the whole brain, smokers have higher levels of Ni, Zn, Cd, La, Pr, Nd, Tb, Tm, Na, Mg, P, K, Fe, Cs, Tl, Cr, and As, and lower concentrations of Al, V, Mn, Co, Cu, Ce, Eu, Gd, Dy, Er, Ca, Rb, Sr, Sb, Ba, Pb, and Se (Figure 7).

**Figure 8:**
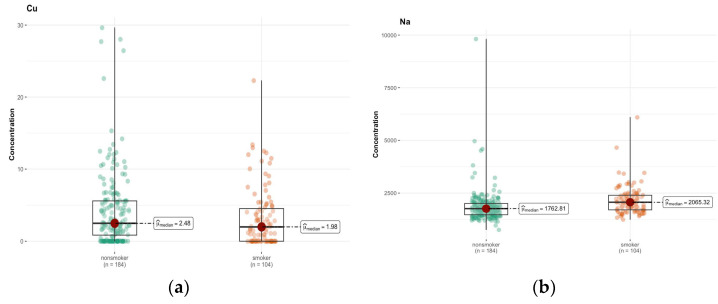
Comparison of difference in median values of Cu (**a**) and Na (**b**) content (µg/g w.w.) in the whole brain tissue of smokers (*n* = 104) and non-smokers (*n* = 184).

An analysis of the results of the Mann–Whitney test shows significant differences in the concentration of some elements in the brain between smokers and non-smokers (Table S2a, Supplementary Materials). In particular, a significantly lower concentration of Cu (*p* = 0.047) was observed in smokers compared to non-smokers (Figure 8a). Another significant result is a higher concentration of Na in smokers (*p* < 0.001) (Figure 8b). The median Na concentration was 1762.815 µg/g (w.w.) in non-smokers and 2065.320 µg/g (w.w.) in smokers, indicating significant differences in the concentration of this element. For the remaining elements, no statistically significant differences were found between smokers and non-smokers.

**Figure 9:**
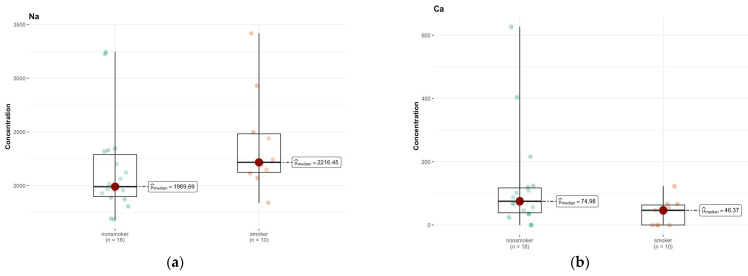
Comparison of difference in median values of Na (**a**) and Ca (**b**) content in the A area of the brain tissue of smokers (*n* = 10), and non-smokers (*n* = 18).

In area A, only calcium (Ca, *p* = 0.057) and sodium (Na, *p* = 0.072) showed *p*-values close to the significance limit, suggesting that their concentration in the brain area might differ between groups (Tables S1f and S2b, Supplementary Materials). However, after Bonferroni correction, these differences were not statistically significant. In the case of calcium, the medians were 74.979 µg/g (w.w.) in non-smokers and 46.372 µg/g (w.w.) in smokers, which may indicate a tendency for lower calcium concentrations in smokers (Figure 9a,b).

**Figure 10:**
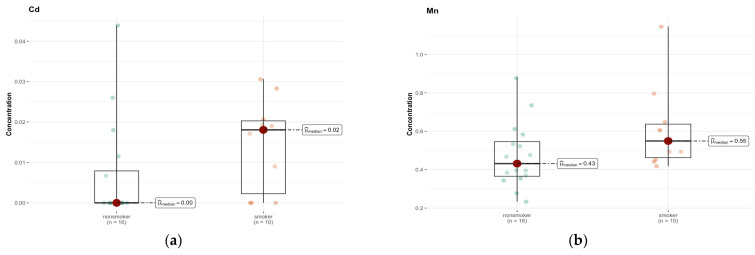
Comparison of difference in median values of Cd (**a**) and Mn (**b**) content (µg/g w.w.) in the B area of the brain tissue of smokers (*n* = 10) and non-smokers (*n* = 16).

In region B, the only elements approaching significance were cadmium (Cd) and Mn (Figure 10a,b). The median is smaller than the limit of detection (DL) (0.00 µg/g; w.w.) for non-smokers and 0.018 µg/g (w.w.) for smokers, indicating a higher concentration of this element in the smokers group (Tables S1g and S2c, Supplementary Materials). The test statistics (46.500) and *p*-value (0.058) suggest a possible difference, but not at the significance level, after Bonferroni correction.

**Figure 11:**
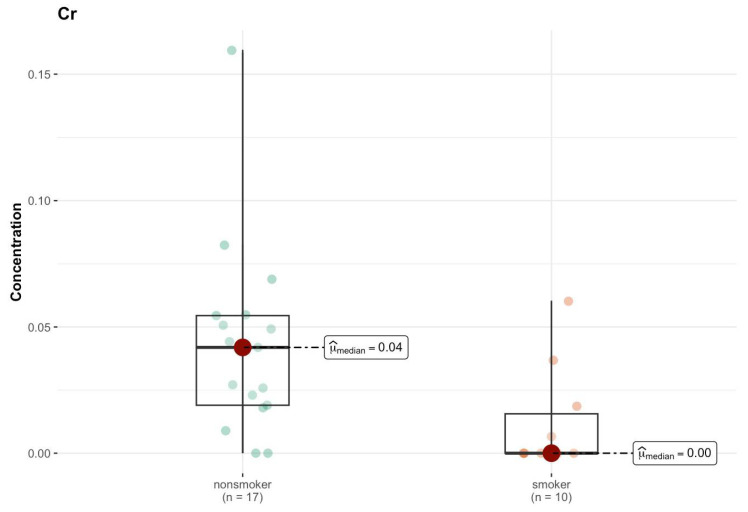
Comparison of statistically significant difference (*p* < 0.01) in median values of Cr content (µg/g w.w.) in C area of the brain tissue of smokers (*n* = 10) and non-smokers (*n* = 17).

An analysis of the results of comparative tests between the group of smokers and non-smokers for individual elements in the C region does not show significant statistical differences for most of the analyzed elements (Tables S1h and S2d, Supplementary Materials). The *p* values, in most cases, exceed the standard significance threshold of 0.05, which suggests a lack of statistically significant differences in the median concentrations of these elements between both groups. The only element for which a significant difference was detected is chromium (Cr) (*p* = 0.010) (Figure 11). Its median concentration was 0.042 µg/g (w.w.) in non-smokers, while in smokers it was <DL (0 µg/g; w.w.), which indicates significantly lower values in this group. The statistical test suggests that this difference is significant and is not due to random variability in the samples.

A comparative analysis of element concentrations between non-smokers and smokers does not show statistically significant differences for most of the elements studied in brain region D (Tables S1i and S2e, Supplementary Materials). The *p* values for all elements analyzed are higher than 0.05, which means that there is not enough evidence to state that smoking affects the concentrations of these elements in the examined brain region. The median concentrations for both groups are very similar, and in many cases identical, which suggests that smoking does not have a significant effect on the level of these elements. Some elements, such as cadmium (Cd) and manganese (Mn), show small differences in median values between groups. For example, the median cadmium concentration in smokers is 0.008 µg/g (w.w.), while in non-smokers it is <DL (0.000 µg/g; w.w.). Similarly, the median manganese concentration in smokers is slightly higher (0.531 µg/g; w.w.) than in non-smokers (0.432 µg/g; w.w.). However, the *p* values for these elements (0.167 and 0.058, respectively) do not reach statistical significance, indicating that these differences may be due to chance.

In the analysis of differences between smokers and non-smokers for various elements in region E, none of the comparisons showed statistical significance after correction for multiple testing (Tables S1j and S2f, Supplementary Materials). Magnesium (Mg) was the only element for which the differences between groups were close to significance (*p* = 0.027), which may suggest potential deviations in its concentration. Non-smokers had a higher median (114.053 µg/g; w.w.) compared to smokers (100.910 µg/g; w.w.).

A statistically significant difference was detected in the level of medians for several elements for which *p* < 0.1 in region F, i.e., Tm (*p* = 0.054) and Ce (*p* = 0.072) (Tables S1k and S2g, Supplementary Materials).

A statistically significant difference was detected in the level of medians for elements for which *p* < 0.1 in region G for Na (*p* = 0.074). The group of smokers contained more sodium (1588.444 µg/g w.w.) compared to non-smokers (1446.051 µg/g; w.w.) (Tables S1l and S2h, Supplementary Materials).

**Figure 12:**
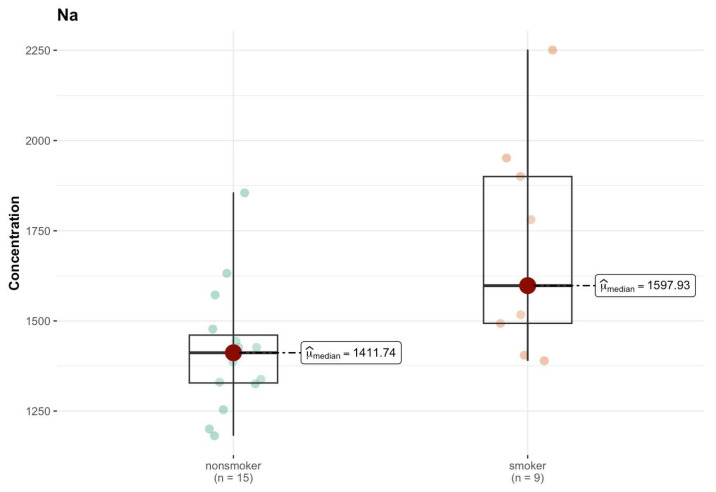
Comparison of statistically significant difference (*p* < 0.01) in median values of Na content (µg/g w.w.) in H area of the brain tissue of smokers (*n* = 9) and non-smokers (*n* = 15).

In region H, the test statistics and *p* values do not suggest significant differences between groups (Tables S1m and S2i, Supplementary Materials). The only element that shows a significant difference between groups is sodium (Na), where *p* = 0.010 indicates a statistically significant higher concentration in the smokers group (median 1597.932 µg/g; w.w.) compared to non-smokers (median 1411.739 µg/g; w.w.) (Figure 12). This may suggest some systematic difference in sodium levels between groups.

**Figure 13:**
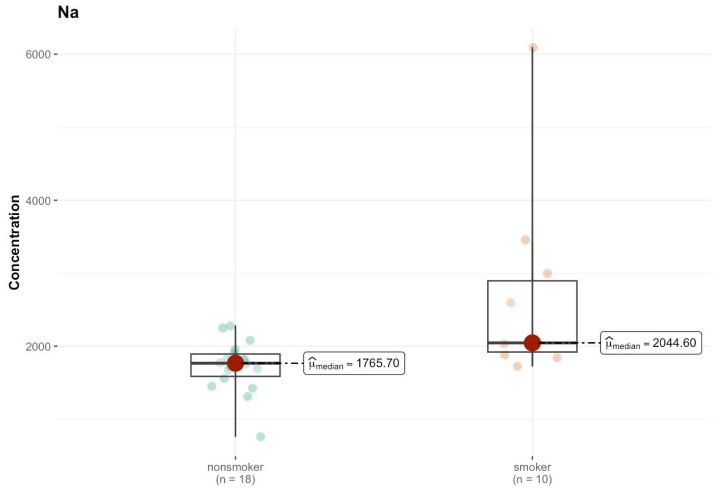
Comparison of statistically significant differences (*p* < 0.05) in median values of Na content (µg/g w.w.) in I area of the brain tissue of smokers (*n* = 10) and non-smokers (*n* = 18).

In area I, the results of the comparison tests for various elements between the group of smokers and non-smokers indicate a lack of statistically significant differences for most of the analyzed elements (Tables S1n and S2j, Supplementary Materials). One of the few elements for which a lower *p* value was observed is sodium (Na), for which the test indicates a *p* value of 0.003, suggesting a statistically significant difference between the groups. The median value of sodium in smokers is higher than that in non-smokers (2044.602 µg/g; w.w. vs. 1765.695 µg/g; w.w.), which indicates this element was present at different levels in the analyzed samples (Figure 13). For lead (Pb), the *p* value of 0.051 is close to the level of significance, which may suggest that the differences in the levels of this element may be significant; however, at the level of 0.05, the result is ambiguous. The medians are low, and amount to 0.038 µg/g (w.w.) for non-smokers and <DL (0.000 µg/g; w.w.) for smokers, which may suggest a reduction in the levels of this element that were detected in the samples of smokers. Similar results were obtained for Ce (*p* = 0.097), where the median in both groups was <DL.

**Figure 14:**
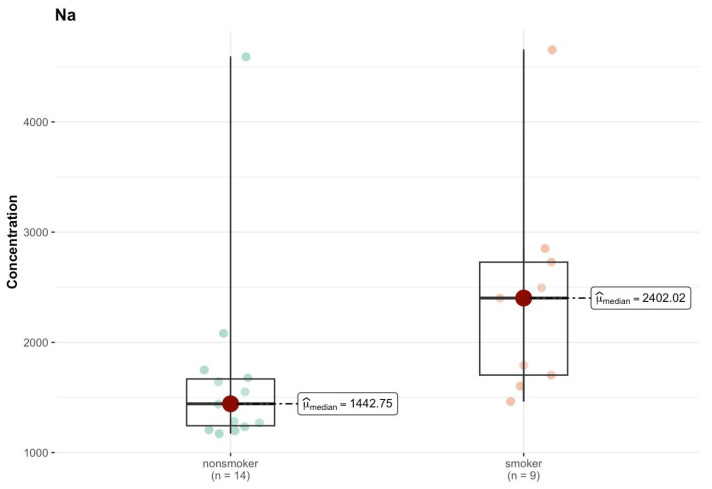
Comparison of statistically significant differences (*p* < 0.05) in median values of Na content (µg/g w.w.) in J area of the brain tissue of smokers (*n* = 9) and non-smokers (*n* = 14).

In the J region, cadmium (Cd) in the non-smokers group had a median of 0.004 µg/g (w.w.), while among smokers this increased to 0.023 µg/g (w.w.) (Tables S1o and S2k, Supplementary Materials). However, the *p*-value of 0.554 indicates that this difference is not statistically significant. Chromium (Cr) had a higher median among smokers (0.034 µg/g; w.w.) compared to non-smokers (0.023 µg/g; w.w.), but the *p*-value (0.544) suggests a lack of statistical significance. For copper (Cu), a difference in the median is observed—3.311 µg/g (w.w.) for non-smokers and <DL (0 µg/g; w.w.) for smokers—which may suggest a difference in the concentration of this element between the groups, but the *p*-value (0.403) indicates that this difference is not significant. Only Na shows a significant difference in the median—1442.755 µg/g (w.w.) in the non-smokers group and 2402.024 µg/g (w.w.) in the smokers group (Figure 14). The *p*-value (0.005) suggests that this difference is statistically significant.

**Figure 15:**
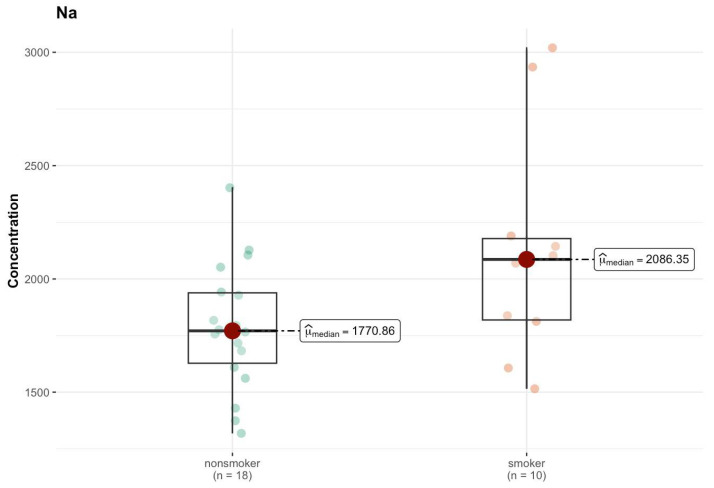
Comparison of difference in median values of Na content (µg/g w.w.) in the K area of the brain tissue of smokers (*n* = 10) and non-smokers (*n* = 18).

In the K region, the aluminum (Al) level showed a significant difference (*p* = 0.030), although both groups had a median smaller than that of DL (0 µg/g; w.w.) (Tables S1p and S2l, Supplementary Materials). This may mean that despite the zero median in both groups, higher concentration values of this element were present in the smokers’ samples, which suggests that smoking influences its presence in the body. For sodium (Na), the median in the smokers’ group was clearly higher (2086.35 µg/g; w.w.) compared to non-smokers (1770.87 µg/g; w.w.), and the test result (*p* = 0.057) was close to the borderline of statistical significance (Figure 15).

### 2.5. Comparison of Elemental Content in Spinal Cord Between Smokers and Non-Smokers

**Figure 16:**
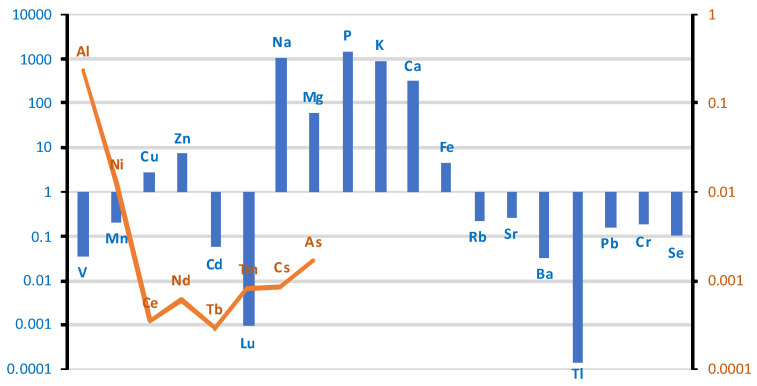
Comparison of differences in mean values of individual elements at a logarithmic scale in spinal cord tissue between groups of smokers and non-smokers. Blue bar represents log(case mean–control mean); orange line represents log(control mean–case mean).

In the case of the spinal cord (Tables S1r and S2m, Supplementary Materials), the distribution of elements according to the median values is presented in Figure 16. The elemental analysis of spinal cord tissue revealed high concentrations (3500–100 µg/g; w.w.) of phosphorus (P), potassium (K), sodium (Na), and magnesium (Mg). Trace elements, including calcium (Ca), iron (Fe), zinc (Zn), rubidium (Rb), and manganese (Mn), were detected within the range of 60–0.6 µg/g (w.w.). Additionally, selenium (Se), strontium (Sr), chromium (Cr), vanadium (V), and cadmium (Cd) were present at concentrations between 0.2 and 0.02 µg/g (w.w.). The elemental distribution reflects the metabolic activity and ionic regulation essential for the function and integrity of neural tissues.

In the case group, the spinal cord tissue contains more V, Mn, Cu, Zn, Cd, Lu, Na, Mg, P, K, Ca, Fe, Rb, Sr, Ba, Tl, Pb, Cr, and Se. Lower concentrations compared to the control group were observed for the following elements: Al, Ni, Ce, Nd, Tb, Tm, Cs, and As. For a large group of elements including Co, La, Pr, Sm, Eu, Gd, Dy, Ho, Er, Yb, Be, B, and Sb, the detected changes were outside the detection limit.

The results of the Mann–Whitney test indicate that only sodium (Na) concentration differs statistically significantly between the smokers and non-smokers (*p* = 0.040). Phosphorus (*p*) (*p* = 0.056) and zinc (Zn) (*p* = 0.065) show *p* values close to the significance threshold, but do not exceed it. These results should be considered as guidelines for further research. Similarly, selenium (Se) (*p* = 0.087), despite its role in neutralizing oxidative stress (related to smoking), did not show a significant difference, although the *p* value suggests a certain trend (Figure S3, Supplementary Materials).

## 3. Discussion

An important aspect of cigarette smoking is the disruption of microelement and trace element homeostasis and the accumulation of toxic elements. Toxic metals, together with other toxins, enter the body through tobacco smoke.

The use of the Mann–Whitney U test in this study was dictated by the characteristics of the data obtained. Elemental concentrations measured in post-mortem human tissues did not follow a normal distribution, as verified through descriptive statistics and exploratory data analysis. These deviations from normality are typical in biological and environmental datasets, especially when the sample size is limited and includes many non-detects or values below the detection limit. Given the non-normal distribution of data and the relatively small sample sizes in both the smoker and non-smoker groups, a non-parametric statistical approach was the most appropriate. The Mann–Whitney U test is a widely used non-parametric alternative to Student’s *t*-test that does not require the assumption of normality or homogeneity of variances. It compares the medians of two independent groups and is suitable for ordinal or continuous data that are skewed or contain outliers. In this study, the Mann–Whitney U test was applied consistently across tissue types to compare the concentrations of individual chemical elements between smokers and non-smokers. The test allowed for the identification of statistically significant differences (e.g., in aluminum, lead, copper, sodium, and thallium levels), while minimizing the risk of erroneous conclusions that might result from applying parametric tests to non-normally distributed data. The decision to use this test enhances the reliability of the findings and ensures their methodological adequacy.

A statistically significantly higher Al content was found in smokers’ bronchial and lung tissue. The scatter in Figure 2 suggests that Al accumulation in the bronchi of smokers may be a function of the length of exposure, type of smoking (cigarettes vs. pipe), and tobacco source. A similar trend was observed for the Tl content in the liver of smokers, as shown in Figure 6c. On the other hand, the concentration of elements such as Cu, Rb, and Se in the bronchial tissue was lower than that in the control tissue, and the *p*-values in these cases were close to the limit of 0.05. It is known that Al is an element that has no physiological function and is toxic even at low concentrations [62]. Se and Cu, on the other hand, are essential trace elements that are responsible for the activity of enzymes that form the body’s antioxidant barrier. Se, as a component of selenoproteins (GPx1, GPx2, GPx4), also plays an antiviral role, and is responsible for male reproductive functions and for maintaining the integrity of membranes in the gastrointestinal tract; and Se-dependent iodothyronine deiodinase enzymes (Dio1, Dio2) are responsible for intrathyroidal iodine metabolism [63,64,65]. Cu deficiency and impaired homeostasis of this element can disrupt many processes that are key to the body’s immune and antioxidant defenses, iron metabolism, neurotransmission, energy metabolism, and erythropoiesis, and negatively affect skeletal development and melanin synthesis [66,67,68]. Therefore, Cu and Se dyshomeostasis, along with other elements such as Zn and Mg, may occur in pathological conditions associated with oxidative stress and inflammation [69,70,71,72,73,74,75].

The biological role of Rb in the human body is not fully understood. Studies suggest that Rb is similar to K and Cs in its behavior in physiological processes. Rb may therefore be involved in K metabolism, especially since Na^+^/K^+^ ATPase has the same affinity for K as Rb [76,77]. In the central nervous system (CNS), Rb increases synaptic dopamine and norepinephrine levels and acts similarly to serotonin. Several experiments in animal models and humans have been described in which the administration of rubidium chloride was found to be as effective as tricyclic antidepressants in the treatment of depressive disorders [78,79]. The problem in diagnosing excess/deficiency is the lack of reference ranges for trace elements such as Rb and the need to use highly specialized analytical techniques for their determination, such as ICP-MS and inductively coupled plasma optical emission spectrometry (ICP-OES). In summary, Rubidium is not a natural element in homo sapiens and is not considered essential to any organism [80]. Although Rb can partially replace K, and there is a possibility that it may have modest beneficial effects under certain conditions, it should be considered a form of toxicity.

A significantly higher Al content was also found in the lung tissue of smokers. There was also an accumulation of rare earth elements, i.e., Ce, Pr, Nd, Pr, Sm, Eu, Dy, and Er. Rare earth elements are poorly understood in terms of their presence in tissues and their potential role in the body. Lanthanides are similar in terms of their physico-chemical properties. The ionic form occurs in oxidation state +3, and less commonly in oxidation states +2 and +4. It is generally believed that these elements have low toxicity and can be used in medicine for diagnosis and therapy. For example, Gd 3+ has been used as a contrast agent in magnetic resonance imaging (MRI) [81,82]. There is even a hypothesis that lanthanides act as Ca analogs in biological systems. Although this hypothesis originated in the last century, further research is needed [83,84,85]. A significantly higher concentration of Dy was observed in the right lung of the study group, and, in addition, statistical significance was achieved in the left lung for Er, Nd, Pr, Sm, and Mn, as well as Na and Sr. There were also many strong positive interelement correlations for Al vs. Pr, Nd, Sm, Er, and Dy. In the group of smokers, the correlations were weaker in the right lung than in the left lung, indicating not only the effect of smoking on the balance of elements in the body, but also the asymmetric distribution of elements in the two lungs.

Liver tissue from smokers contained significantly more toxic Pb and Tl and less Cu than the control group. A strong K-Rb correlation was observed in the non-smoker group (r = 0.81, CI = [0.53, 0.93], *p* = 0.04), confirming the similarity described above in terms of the role these elements play in physiological processes. However, most of the observed correlations concern rare earth elements, the role of which is poorly understood. The increased accumulation of Pb, one of the most toxic heavy metals, may be of concern [86]. Many studies confirm that most Pb accumulates in the liver [87,88]. The toxicity of Pb is due to the generation of oxidative stress and the depletion of antioxidant systems, resulting in damage [89].

This observation may explain a report published in *Nature* showing a high probability of developing non-alcoholic fatty liver disease as a result of smoking [90] and other reports linking smoking with an increased risk of fibrosis and the development of hepatocellular carcinoma [91].

At the same time, Cu levels were significantly lower in the livers of smokers. Previously published reports refer to the level of Cu in body fluids, and smokers were found to have lower urinary copper concentrations [92], while plasma copper concentrations were significantly higher in smokers than in non-smokers (120 ± 19 vs. 100 ± 16 μg/dL, *p* < 0.01 [93,94].

There were no significant differences in the spinal cord (apart from a higher Na content in the smokers group), but the presence of a higher concentration of toxic elements Pb, Cr, Al, and Cd in the smokers group is worth noting.

The smoker’s brain contained significantly more Na and less Cu compared to non-smokers. Studies to date on the effects of smoking on Na levels in the body have yielded inconsistent results. Most studies indicate that smokers may have higher serum Na levels than non-smokers. Other authors point to the fact that smoking is associated with a greater preference for salty foods and potentially increased Na intake [95]. The difference in Na levels between smokers and non-smokers in brain tissues may also be due to other reasons. Na is highly regulated in all tissues and is abundant, at ~1200 ug/g in adult brains [96]. Figure 8b and Figure 14 show higher levels of Na spreading in smokers than in non-smokers. These higher Na levels may be due to smoking-related vascular disorders, high blood pressure, poor circulation, and stroke [97]. Analyzing individual areas, it was possible to notice that they had a similar chemical composition, and the differences between groups rarely reached statistical significance. Cd accumulated in areas B and J, and aluminum in area K (insula). The insula is part of the cerebral cortex and is located in the lateral sulcus depression. This is an area that is responsible for understanding human behavior, considered the center of our emotions, intuition, and empathy, but it also participates in the perception of taste and smell, regulates the work of internal organs (blood pressure, heart rate), and integrates the functions of the vestibular system. There are indications that the insula is involved in the existence of addictions. In area B (precentral gyrus), there was more As, Cd, and Mn in the brains of smokers compared to non-smokers, but statistical significance was not reached. The precentral gyrus is part of the primary motor cortex. This part of the brain is responsible for the control of voluntary movements; therefore, any damage to the precentral gyrus affects the upper motor neurons. The location of the precentral gyrus above the pyramidal chiasm causes motor dysfunctions, i.e., muscle weakness, especially distal ones, pathological reflexes (Babinski’s sign), and increased muscle tone with spasticity, to appear on the opposite side of the body [98].

This study investigated the elemental composition of approximately 400 human tissue samples collected from smokers and non-smokers, focusing on the quantification of 41 chemical elements using advanced analytical methodologies. Macroelements P, K, Na, Mg, and Ca were consistently among the most abundant across all tissues. The spinal cord and brain exhibited the highest macroelement concentrations. Microelements Fe and Zn were present across all tissues; Rb and Mn also appeared frequently, with Cu and Se being particularly prominent in liver and brain tissues. Toxic Elements Pb and Cd were detected in all tissues. Notably, brain tissues showed higher levels of Pb and Al, with the additional detection of Ba, Cs, and As. These findings align with earlier reports associating cadmium and aluminum exposure with neurodegenerative diseases and cognitive impairments [99], but uniquely highlight smoking as a significant source of metal accumulation within neural tissues. Smokers exhibited markedly higher concentrations of aluminum (Al) in bronchial and pulmonary tissues. Furthermore, liver samples from smokers demonstrated elevated levels of lead (Pb), thallium (Tl), and rare earth elements (REEs). These findings highlight the distinct elemental profiles of different tissues, reflecting their physiological roles and exposure risks. It can be concluded that the systemic impact of tobacco smoke exposure promotes the bioaccumulation of toxic metals and trace elements across critical organs, underscoring the potential long-term health risks associated with smoking. The accumulation of toxic elements in neurologically sensitive tissues such as the brain and spinal cord suggests potential environmental and health concerns that merit further research.

The study is limited by the relatively small population included in the study. Therefore, in addition to statistically significant differences between the levels of the elements in the groups being compared, the trends of changes deserve attention, especially those close to the significance level. In addition, the results obtained refer only to the inhabitants of south-eastern Poland, which is associated with difficult-to-determine exposure to various environmental toxins and different available brands of cigarettes. The high variability of the measured values, expressed by the SD, as well as the high kurtosis, indicate the presence of outliers for many elements.

## 4. Materials and Methods

### 4.1. Studied Population and Sample Characterization

Tissue samples were collected during autopsies performed at the request of the prosecutor at the Department of Forensic Medicine of the Medical University of Lublin. Consent to tissue collection was approved by the Bioethics Committee of the Medical University of Lublin (approval number KE-0254/181/2021). According to Polish law, securing biological material during forensic autopsies requires the consent of the prosecutor supervising the proceedings. Such a decision is not dependent on obtaining additional consent from the family members of the deceased person. All the samples were obtained from cadavers during a medicolegal autopsy performed no later than 24–48 h after death. All of them were victims of sudden death (mors subita) after road accidents and suicides.

The demographic characteristics of the study population are summarized in Table 4. Fisher’s exact test statistic (0.2264) indicated that the gender distribution between groups was not statistically significant. The Mann–Whitney U test was used to compare mean age, weight, and BMI values between groups. Statistical significance was set at *p* < 0.05.

The research material was collected during autopsies from cerebral hemispheres (*n* = 28), liver (*n* = 26), bronchi (*n* = 27), lungs (*n* = 55), and spinal cord (*n* = 23). Brain samples were collected from the following areas: A—frontal pole (*n* = 28); B—precentral gyrus (*n* = 26); C—postcentral gyrus (*n* = 27); D—cingulate gyrus (*n* = 26); E—hippocampus (*n* = 25); F—head of caudate nucleus (*n* = 26); G—superior longitudinal fasciculus of brain, SLF (*n* = 27); H—inferior longitudinal fasciculus of brain, ILF (*n* = 27); I—dorsal thalamus (*n* = 28); J—nucleus accumbens septi, NAc (*n* = 26); K—insula (*n* = 28).

Belonging to the study/control (smoker/non-smoker) group was determined based on post-mortem documentation in accordance with the established criteria. The study/control group was selected based on the absence/presence of signs of smoking addiction resulting from documentation and a macroscopic evaluation of features attributed to tobacco smokers.

**Figure 17:**
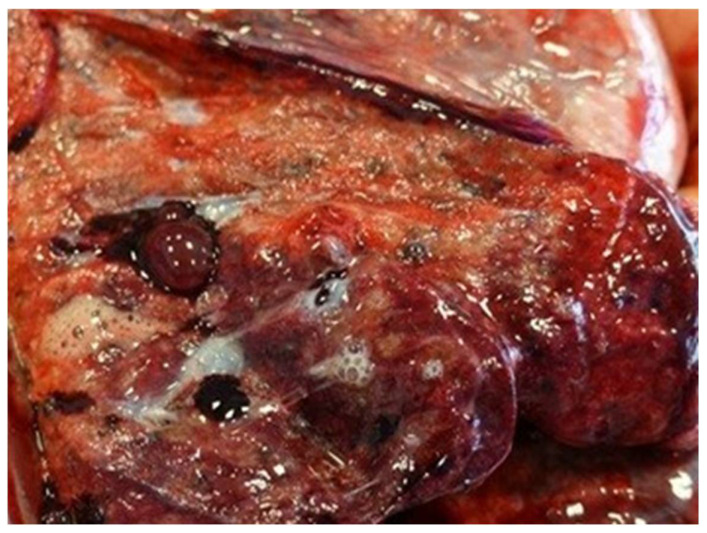
Pathological changes observed in Smoking-Related Interstitial Fibrosis (SRIF).

Lung tissue damage includes smoking-related interstitial lung diseases (SR-ILD), i.e., interstitial lung damage, edema, inflammation, idiopathic pulmonary fibrosis (IPF), diaphragm muscle atrophy, lobular emphysema, combined pulmonary fibrosis and emphysema (CPFE), and lung cancer, which belong to a wide range of characteristic abnormalities and clinical pictures observed in the respiratory system of tobacco smokers [100]. Smoking-related interstitial fibrosis (SRIF) (Figure 17) is a common and morphologically characteristic finding in the lung tissue of cigarette smokers. It can be distinguished histologically from idiopathic interstitial pneumonias and other causes of interstitial lung fibrosis. SRIF is characterized by dense thickening of the alveolar septa by thick collagen bundles with a glassy appearance, with a common admixture of hyperplastic smooth muscle bands [101].

**Figure 18:**
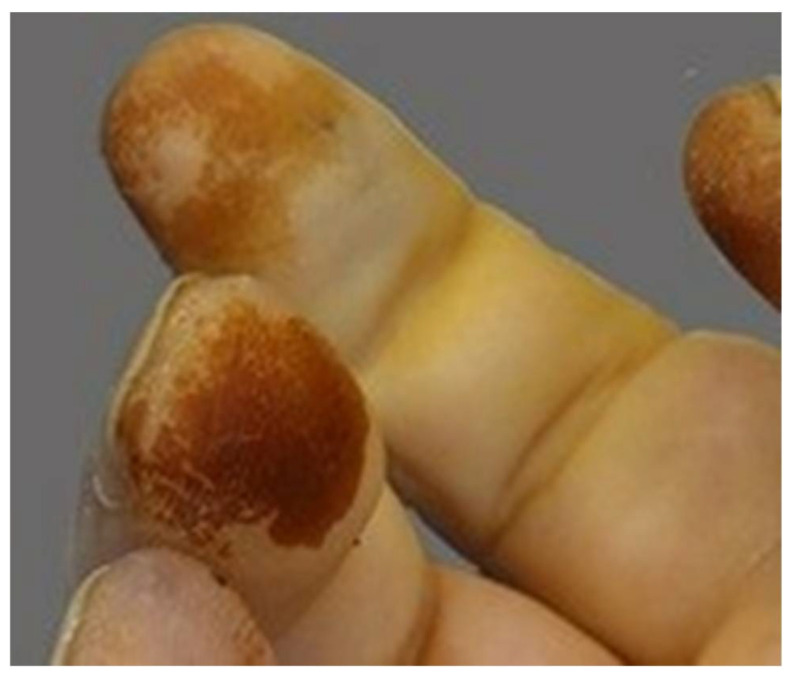
Yellowing of a smoker’s fingers.

Macroscopic assessment of the skin includes features characteristic of smokers, such as premature aging, yellowing of the fingers and nails (Figure 18), and fat accumulation around the waist and upper trunk, with less around the hips [102,103].

**Figure 19:**
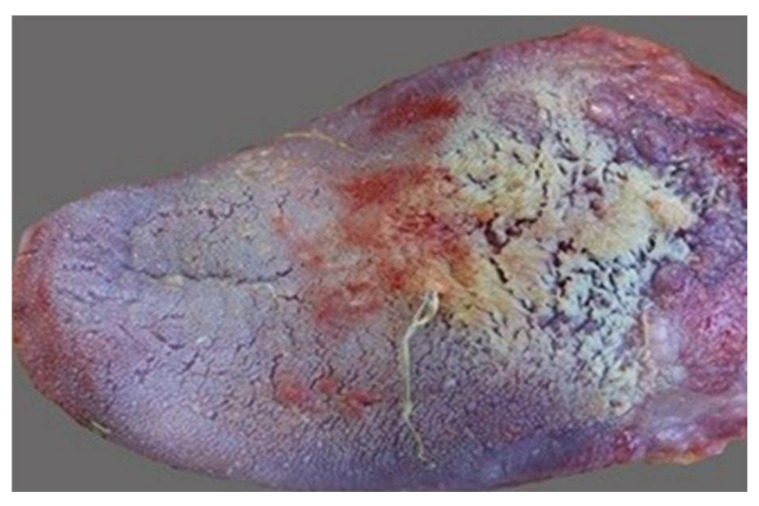
Smoker’s tongue.

Oral conditions in smokers include darker pigmentation of the gingiva (“smoker’s melanosis”); lingual leukoplakia (“smoker’s tongue”, Figure 19), characterized by white spots or patches on the tongue or vulva; and a grayish-white palate with red nodules (nodules) signifying inflammation of the salivary glands (“smoker’s palate”/nicotine stomatitis) [104].

Regarding blood and urine alcohol levels in the case group, 3 out of 10 individuals exhibited blood alcohol levels ranging from 0.82 to 5.07, while in the control group, 8 out of 16 individuals had blood alcohol levels ranging from 0.63 to 3.23. The urine alcohol levels recorded were 2.57.

### 4.2. Sample Collection Procedure

Tissue samples were collected by qualified pathologists. Samples weighing 0.5–1.5 g were rinsed with ultrapure water (Milli-Q, Millipore, Raleigh, NC, USA; resistivity: 18.2 MΩ cm), dried on a sterile filter paper, weighed on an analytical balance, and stored in decontaminated polypropylene tubes at −80 °C until further analysis. Instruments (knives, forceps and scissors) used during autopsy were immersed in 10% HNO_3_ and then rinsed with ultrapure deionized water to minimize the likelihood of sample contamination.

### 4.3. Sample Preparation

Tissue samples were predigested with 7 mL of 69% Suprapur Nitric Acid 65% HNO_3_ (Baker, Radnor, PA, USA) and 1 mL of deionized water. Closed digestion was performed in closed Teflon containers using a microwave digester using a Mars 6 microwave system (CEM, Matthews, NC, USA). Microwave digestion was performed according to the program: 20 min at maximum temperature 185 °C, with a rise time of 10 min and holding time of 10 min. After digestion, the digests were quantitatively transferred to sterile Falcon polypropylene conical tubes with Plug-Seal caps and 1 mL of 35% HCl ultrapure for trace metal analysis (Baker, Radnor, PA, USA) was added to stabilize some elements (As, Se, Mo, Tl). The sample was then diluted to a total volume of 25.0 mL by ultrapure water (>18.2 MΩ cm at 25 °C) obtained via the Milli-Q purification system (Millipore, Darmstadt, Germany). A sample without tissue was used as a control.

### 4.4. Measurements Using ICP MS

All samples were analyzed using ICP MS (PQ MS Q, Analytik Jena, Germany), which is equipped with Integrated Collision-Reaction Cell (iCRC). All detailed operating parameters are provided in the Supplementary Material (Table S1, Supplementary Materials). Multiple certified reference materials (CRMs) were used to validate the ICP MS method (Table S3, Supplementary Materials). The selected CRMs, representing a similar matrix, were used for quality control of the analysis process, e.g., DB001 (human hair), ERM BB 184 (bovine muscle), and BCR 185R (bovine liver), achieving acceptable recovery (80–120%) for most elements (Table S4, Supplementary Materials). For elements with uncertified content, the standard addition method was additionally used. An internal standard of 5 ug L^−1^ (Li6, Sc, Y, Rh, Ir, Bi) was added to all solutions, being pumped to the nebulizer through the Y-shape connector by a separate channel of the spectrometer peristaltic pump. The multielement internal standard allowed for control of both the sample introduction system and the mass analyzer and detection system. Table S5 (Supplementary Materials) presents the DL values for chemical elements measured by ICP MS.

### 4.5. Statistical Analysis

Descriptive statistics were calculated for all tissues examined separately for the control and studied groups. All elements were characterized by right-sided asymmetry in terms of concentration, so the *t*-test could not be used to test the significance of differences between groups, as this requires a normal probability of distribution in both groups, especially when groups are relatively small.

Given the clear deviations from normality, as indicated by the high skewness and kurto-sis values, nonparametric methods were used to verify mutual differences between groups [105]. The Mann–Whitney U test, also known as the Wilcoxon rank-sum test applied in the article, is a non-parametric statistical method used to compare differences between two independent groups when the assumption of normal distribution is not met [106,107]. This test is particularly useful in analyzing data that is skewed, ordinal, or has unequal variances, making it a robust alternative to the traditional *t*-test. The Mann–Whitney U test operates by ranking all data from both groups together; it then calculates the sum of ranks for each group and uses these rank sums to evaluate the differences between the groups. The primary hypothesis tested by the Mann–Whitney U test is whether one group tends to have higher or lower values than the other, without assuming any specific distributional form for the data. This makes the Mann–Whitney U test highly applicable in medicine, psychology, and environmental science, where data may not typically conform to normal distributions due to natural variability, measurement methods, or sample sizes.

The Mann–Whitney U test is robust to nonnormality and small sample sizes [108]. Bonferroni correction was applied to adjust for multiple comparisons and control the family-wise error rate, thus ensuring that significant results were not artifacts of multiple hypothesis testing [109]. *p* < 0.05 was considered as statistically significant.

Box and whisker plots were used to visually compare median values, interquartile ranges, and potential outliers, facilitating the identification of trends and variability between groups [110]. To examine patterns of element concentrations in smokers and non-smokers, PCA analysis was performed as a dimensionality reduction technique [111]. PCA analysis was performed on standardized data to ensure the equal weighting of variables, taking into account the different values of element concentrations [112,113].

## 5. Conclusions

Cigarette smoking remains a major source of exposure to toxic elements, contributing to the accumulation of heavy metals in vital organs. This study provides the first comprehensive analysis of 41 chemical elements in the post-mortem tissues of chronic smokers, revealing significant alterations in essential metals’ homeostasis and the accumulation of toxic elements. The deposition of Al, Pb, and Tl in the bronchi, lungs, and liver highlights the long-term toxic effects of smoking and possibly the induction of oxidative stress, ultimately leading to organ damage and linking smoking to an increased risk of liver or lung fibrosis. Once in the bloodstream, metals are systemically transported and can accumulate in secondary target organs in the central nervous system, accumulating Cd in selected brain regions. The findings emphasize the need for regular monitoring of tobacco products for toxic elements and further research into the mechanisms of their accumulation and potential health effects.

## Figures and Tables

**Table 1:** Comparison of median chemical element concentrations (µg/g; w.w.) in bronchial tissue of non-smokers (1) (*n* = 16) and smokers (2) (*n* = 11) using the Mann–Whitney test. The “statistic” column refers to the Mann–Whitney U test statistic. The notations for statistical significance are as follows: *p* < 0.01 with two asterisks (**).

Element	Min (1)	Max (1)	Min (2)	Max (2)	Median (1)	Median (2)	Statistic	*p*
Al	<DL	<DL	<DL	3.0488	<DL	<DL	48.000	0.004 **
As	<DL	0.0159	<DL	0.0183	<DL	<DL	94.500	0.701
Ba	<DL	0.1443	<DL	0.087	<DL	<DL	71.000	0.188
Ca	28.6072	2237.151	139.8396	4498.682	400	330	78.000	0.645
Cd	<DL	0.1855	0.0103	0.2513	0.037	0.043	87.000	0.980
Ce	<DL	0.0098	<DL	0.0096	<DL	0.003	65.500	0.261
Co	<DL	0.032	<DL	0.011	0.002	0.004	67.000	0.308
Cr	<DL	0.331	<DL	0.04	0.016	0.009	101.500	0.502
Cs	<DL	0.007	<DL	0.0061	<DL	<DL	100.000	0.533
Cu	<DL	5.7606	<DL	1.0924	0.54	0.19	127.000	0.057
Dy	<DL	0.0005	<DL	<DL	<DL	<DL	93.500	0.451
Fe	12.1397	76.2908	4.6835	60.2807	27	22	92.000	0.865
Gd	<DL	0.0403	<DL	0.0024	<DL	<DL	91.000	0.821
K	781.3791	2806.727	764.645	2006.422	1300	1200	103.000	0.481
La	<DL	0.0045	<DL	0.0433	<DL	<DL	85.000	0.786
Mg	65.9929	236.6223	81.2823	271.2504	110	110	88.000	1.000
Mn	<DL	0.9565	0.122	0.8899	0.27	0.22	94.000	0.786
Na	1256.017	3442.592	1743.74	3130.921	2100	2000	82.000	0.790
Nd	<DL	0.0021	<DL	0.0014	<DL	<DL	97.500	0.472
Ni	<DL	0.5856	<DL	0.2636	0.006	0.002	95.000	0.740
P	552.3	4377.063	575.9288	4336.314	880	810	80.000	0.716
Pb	<DL	0.4585	<DL	0.2984	0.041	0.027	83.000	0.824
Pr	<DL	0.0026	<DL	0.0006	<DL	<DL	86.000	0.871
Rb	0.8229	2.8272	0.4701	2.7236	1.6	1.3	122.000	0.099
Se	0.1024	0.2518	0.079	0.215	0.15	0.13	120.000	0.121
Sr	0.0724	3.1352	0.3364	7.6229	0.58	0.68	82.000	0.790
Tb	<DL	0.0022	<DL	0.003	<DL	0.001	62.000	0.146
Tl	<DL	0.0014	<DL	0.001	<DL	<DL	107.500	0.245
Tm	<DL	0.0156	<DL	<DL	<DL	<DL	99.000	0.254
V	<DL	0.0857	<DL	0.0588	0.008	<DL	98.000	0.619
Zn	3.4497	14.7706	4.1378	13.8104	6.9	8.1	77.000	0.610

**Table 2:** Comparison of median chemical element concentrations (µg/g; w.w.) in left and right lung tissue of smokers and non-smokers, analyzed by Mann–Whitney U test. *p* < 0.05 is marked with one asterisk (*).

Left Lung; 1—Non-Smokers; 2—Smokers *n*1 = 17, *n*2 = 11					Right Lung, 1—Non-Smokers; 2—Smokers *n*1 = 16, *n*2 = 11			
Element	Median 1 Min–Max	Median 2 Min–Max	Statistic	*p*	Median 1 Min–Max	Median 2 Min–Max	Statistic	*p*
Al	3.9 0–66.2991	13 1.0861–81.6244	45	0.022 *	2.9 0.9809–16.3328	10 0–34.1825	43	0.026 *
As	ND	ND	-	-	<DL 0–0.0093	<DL 0–0.0095	85	0.786
Ba	<DL 0–0.0572	<DL 0–0.189	73	0.122	ND	ND	-	-
Be	<DL 0–0	<DL 0–0.0022	85	0.242	ND	ND	-	-
Ca	61 0–152.1785	130 0–2121.157	53.5	0.063	66 0–146.2553	64 17.0846–493.4554	80	0.711
Cd	0.11 0.0056–0.4501	0.046 0.0099–0.1428	113	0.378	0.072 0–0.6907	0.036 0–0.1428	116.5	0.167
Ce	0.010 0–0.0656	0.016 0–0.1181	67.5	0.23	0.011 0.0019–0.0535	0.010 0–0.051	85	0.904
Co	0.004 0–0.0146	0.005 0–0.0164	78	0.48	0.004 0–0.0109	0.004 0–0.0092	90.5	0.921
Cr	0.045 0–0.2734	0.11 0–0.6297	54	0.066	0.050 0.0208–0.2383	0.056 0.0089–0.3173	83.5	0.844
Cs	<DL 0–0.0092	0.003 0–0.0165	60	0.106	0.001 0–0.0079	0.004 0–0.0103	72	0.423
Cu	0.26 0–1.3773	0.44 0–4.0041	63	0.157	0.28 0–2.4577	0.46 0–4.2812	71	0.413
Dy	<DL 0–0.0016	0.0003 0–0.0023	57	0.041 *	<DL 0–0.0005	<DL 0–0.0011	54.5	0.035 *
Er	<DL 0–0.0118	0.001 0–0.0148	42	0.003 *	<DL 0–0.0011	<DL 0–0.0035	64.5	0.119
Eu	<DL 0–0.001	<DL 0–0.0004	91	0.833	<DL 0–0.0012	<DL 0–0.0012	85.5	0.828
Fe	160 30.1028–282.943	150 60.6105–250.6031	91	0.926	180 69.3616–297.2741	180 15.9543–323.7149	92	0.865
Gd	<DL 0–0.0148	<DL 0–0.0029	96	0.861	<DL 0–0.0023	<DL 0–0.0022	86	0.871
K	1500 753.474–2301.703	1700 878.5678–2001.746	96	0.926	1600 690.7442–2264.628	<DL 580.8769–1961.902	82	0.79
La	<DL 0–0.4883	<DL 0–0.0553	77	0.322	<DL 0–0.0895	<DL 0–0.0171	89.5	0.942
Mg	69 40.771–86.6753	73 32.7442–126.2084	75	0.404	62 41.498–90.4246	65 40.82–82.992	71	0.422
Mn	0.18 0.0688–1.609	0.31 0–0.4757	51	0.047 *	0.15 0–0.4211	0.22 0.087–0.7414	50	0.064
Na	1300 821.2906–1822.073	1500 1063.692–2093.654	50	0.042 *	1300 923.6779–2391.144	1400 631.091–1823.976	82	0.79
Nd	<DL 0–0.017	0.005 0–0.0351	45.5	0.018 *	0.001 0–0.0118	0.002 0–0.0201	71	0.394
Ni	0.006 0–0.1896	0.020 0–0.0492	85	0.698	0.003 0–0.4607	0.004 0–0.0679	83.5	0.836
P	1000 620.7558–1645.799	1100 616.3822–2694.085	81	0.578	1000 574.577–1337.523	940 583.7071–1451.195	86	0.942
Pb	0.013 0–0.1275	0.041 0–0.1473	57	0.089	0.009 0–0.522	0.014 0–0.0853	62	0.2
Pr	<DL 0–0.0021	0.001 0–0.0112	40.5	0.004 *	<DL 0–0.0172	0.0005 0–0.001	67	0.256
Rb	2.0 0.8012–2.7632	1.7 0.6281–2.6911	123	0.175	1.8 0.7179–2.4813	1.5 0.418–2.3999	104	0.451
Sb	<DL 0–0.0107	<DL 0–0.0301	81	0.293	<DL 0–0.0231	<DL 0–0.0335	85	0.786
Se	0.18 0.0989–0.5166	0.16 0.0849–0.2538	107	0.547	0.16 0.1169–0.2986	0.14 0.0842–0.2203	116	0.178
Sm	<DL 0–0	<DL 0–0.0049	59.5	0.01 *	ND	ND	-	-
Sr	0.22 0.1029–1.1926	0.58 0.1686–1.2492	40	0.011 *	0.23 0.0556–1.2585	0.24 0.0262–1.5324	79	0.68
Tb	<DL 0–0.0012	<DL 0–0.0008	94	1	<DL 0–0.0041	<DL 0–0.0014	88	1
Tl	<DL 0–0.0013	0.0003 0–0.0019	66.5	0.146	<DL 0–0.0025	0.0004 0–0.0022	68.5	0.312
Tm	<DL 0–0	<DL 0–0.0027	85	0.242	ND	ND	-	-
V	0.030 0–0.085	0.023 0–0.1738	85	0.705	0.025 0–0.1068	0.020 0–0.0578	100	0.567
Zn	8.0 5.4195–16.3532	9.0 3.7135–12.9737	70	0.285	7.9 4.9786–11.2627	7.2 5.6125–13.3734	92	0.865

**Table 3:** Comparison between the elemental liver content (µg/g; w.w.) of non-smokers (1) (*n* = 17) and smokers (2) (*n* = 9) using the Mann–Whitney test. *p* < 0.05 is marked with one asterisk (*).

Element	Min 1	Max 1	Min 2	Max 2	Median1	Median 2	Statistic	*p*
Al	<DL	9.6075	<DL	0.7099	<DL	<DL	84	0.629
As	<DL	0.0115	<DL	0.0109	<DL	<DL	72	0.754
Ca	<DL	87.5888	<DL	63.3913	41	39	78	0.957
Cd	0.0552	1.5265	0.1009	0.8344	0.23	0.21	79	0.916
Ce	0.0013	0.1003	<DL	0.0346	0.010	0.005	103.5	0.153
Co	<DL	0.032	0.0077	0.0188	0.008	0.012	48.5	0.138
Cr	<DL	0.0745	<DL	0.0729	0.018	0.017	93	0.388
Cs	<DL	0.0199	0.0042	0.0204	0.008	0.007	75	0.957
Cu	<DL	15.873	<DL	4.9786	4.1	2.0	116.5	0.033 *
Fe	39.952	727.4559	69.35	370.176	130	160	65	0.56
Gd	<DL	0.2612	<DL	0.0146	<DL	<DL	73	0.726
K	1483.552	3544.808	1362.333	2807.976	2400	2200	106	0.12
La	<DL	0.0141	<DL	0.058	<DL	<DL	72	0.754
Mg	101.1198	217.3327	109.5691	163.2676	150	130	101	0.2
Mn	1.118	6.5598	2.4224	5.1005	3.1	3.6	64	0.525
Na	758.9443	2813.448	1049.011	1839.927	1100	1300	53	0.22
Nd	<DL	0.0081	<DL	0.0028	<DL	<DL	94	0.28
Ni	<DL	0.5249	<DL	0.365	0.001	0.008	74	0.91
P	1887.783	3967.537	1933.888	2731.597	2300	2400	82	0.792
Pb	<DL	0.2863	0.0685	0.7855	0.054	0.11	35	0.025 *
Pr	<DL	0.0027	<DL	0.0011	<DL	<DL	85	0.581
Rb	1.2687	6.9822	1.453	4.513	3.7	2.9	108	0.095
Se	0.2318	2.0931	0.2941	0.643	0.44	0.39	93	0.396
Sm	<DL	0.0012	<DL	<DL	<DL	<DL	81	0.518
Sr	<DL	0.7505	<DL	0.2441	0.13	0.065	90.5	0.466
Tb	<DL	0.0041	<DL	0.0024	<DL	<DL	70	0.679
Tl	<DL	0.0009	<DL	0.0012	<DL	<DL	47.5	0.025 *
Tm	<DL	0.0083	<DL	<DL	<DL	<DL	85.5	0.322
V	<DL	0.0355	<DL	0.0332	<DL	0.011	64	0.482
Zn	13.1965	178.2931	21.0617	77.3686	40	30	107	0.107

**Table 4:** Demographic characteristics of the patient groups enrolled in the study.

Group	Controls (*n* = 17)	Cases (*n* = 11)	*p*
Mean age ± SD ^2^	54.50 ± 17.99	56.01 ± 14.04	0.8172
Gender (*n*%)	female: 8 (47.06%)	female: 2 (18.18%)	0.2264
	male: 9 (52.94%)	male: 9 (81.81%)	
Mean weight [kg]	69.13 ± 20.18	76.82 ± 14.44	0.2592
BMI ^1^ [kg m^−2^] (mean ± SD ^2^)	23.69 ± 6.03	24.84 ± 3.75	0.5464

Abbreviations: ^1^. Body mass index (BMI); ^2^. the standard deviation (SD).

## Data Availability

The original contributions presented in this study are included in the article/Supplementary Material. Further inquiries can be directed to the corresponding authors.

## Supplementary Materials

The following supporting information can be downloaded at: https://www.mdpi.com/article/10.3390/ijms26136368/s1.

## Abbreviations

The following abbreviations are used in this manuscript:

CNS	The central nervous system
COPD	Chronic obstructive pulmonary disease
CPFE	Combined pulmonary fibrosis and emphysema
DL	Limit of detection
FDA	The US Food and Drug Administration
HPT	Heated tobacco products
GYTS	Global Youth Tobacco Survey
iCRC	Integrated collision–reaction cell
SRIF	Smoking-related interstitial fibrosis
NHANES	The National Health and Nutrition Examination Survey
ICP-OES	Inductively coupled plasma optical emission spectroscopy
MRI	Magnetic resonance imaging
PCA	Principal component analysis
SLF	Superior longitudinal fasciculus of brain
ILF	Inferior longitudinal fasciculus of brain
NAc	Nucleus accumbens septi
SR-ILD	Smoking-related interstitial lung diseases
IPF	Idiopathic pulmonary fibrosis
ICP-MS	Inductively coupled plasma mass spectrometry
WHO	World Health Organization
w.w.	Wet weight
