# Supplementary material for: Evidence of Organ-Specific Metal Accumulation: ICP-MS Elemental Analysis of Autopsy Tissues of Tobacco Smokers
Source: Int J Mol Sci. 2025 Jul 2;26(13):6368. doi: 10.3390/ijms26136368 (PMC12249502; doi:10.3390/ijms26136368)
Supplement: Supplementary file 1 [file ijms-26-06368-s001.zip › ijms-3673626-supplementary.pdf]

Table S1

(a) Descriptive statistics of element contents in bronchial tissue for the entire study population (n=27).

element	n	Mean	SD	Median	MAD	Min	Max	Skewness	Kurtosis
Al	27	0.26	0.67	0.00	0.00	0.00	3.05	3.30	12.13
As	27	0.00	0.01	0.00	0.00	0.00	0.02	1.81	2.04
B	27	0.00	0.00	0.00	0.00	0.00	0.00	NaN	NaN
Ba	27	0.01	0.03	0.00	0.00	0.00	0.14	2.79	7.69
Be	27	0.00	0.00	0.00	0.00	0.00	0.00	NaN	NaN
Ca	27	967.12	1231.50	335.20	269.63	28.61	4498.68	1.98	3.25
Cd	27	0.06	0.06	0.04	0.02	0.00	0.25	2.24	5.54
Ce	27	0.00	0.00	0.00	0.00	0.00	0.01	1.26	1.17
Co	27	0.00	0.01	0.00	0.00	0.00	0.03	3.51	15.10
Cr	27	0.02	0.06	0.01	0.02	0.00	0.33	4.79	24.04
Cs	27	0.00	0.00	0.00	0.00	0.00	0.01	0.80	−1.12
Cu	27	0.91	1.52	0.24	0.36	0.00	5.76	2.46	5.75
Dy	27	0.00	0.00	0.00	0.00	0.00	0.00	5.20	27.00
Er	27	0.00	0.00	0.00	0.00	0.00	0.00	NaN	NaN
Eu	27	0.00	0.00	0.00	0.00	0.00	0.00	NaN	NaN
Fe	27	28.43	16.10	24.56	12.70	4.68	76.29	1.33	1.96
Gd	27	0.00	0.01	0.00	0.00	0.00	0.04	5.16	26.71
Ho	27	0.00	0.00	0.00	0.00	0.00	0.00	NaN	NaN
K	27	1372.42	496.95	1262.46	309.24	764.64	2806.73	1.38	1.82
La	27	0.00	0.01	0.00	0.00	0.00	0.04	5.11	26.35
Lu	27	0.00	0.00	0.00	0.00	0.00	0.00	NaN	NaN
Mg	27	128.40	52.79	102.58	31.58	65.99	271.25	1.25	0.90
Mn	27	0.32	0.25	0.24	0.16	0.00	0.96	1.32	1.29
Na	27	2174.04	587.03	1995.71	269.22	1256.02	3442.59	0.94	0.32
Nd	27	0.00	0.00	0.00	0.00	0.00	0.00	2.28	3.93
Ni	27	0.04	0.12	0.00	0.00	0.00	0.59	4.15	17.90
P	27	1409.49	1179.11	844.32	388.19	552.30	4377.06	1.78	2.07
Pb	27	0.09	0.13	0.04	0.06	0.00	0.46	1.86	2.72
Pr	27	0.00	0.00	0.00	0.00	0.00	0.00	4.53	21.31

element	n	Mean	SD	Median	MAD	Min	Max	Skewness	Kurtosis
Rb	27	1.53	0.63	1.43	0.71	0.47	2.83	0.42	−0.33
Sb	27	0.00	0.00	0.00	0.00	0.00	0.00	NaN	NaN
Se	27	0.15	0.05	0.14	0.04	0.08	0.25	0.80	−0.19
Sm	27	0.00	0.00	0.00	0.00	0.00	0.00	NaN	NaN
Sr	27	1.44	1.58	0.68	0.51	0.07	7.62	2.55	8.49
Tb	27	0.00	0.00	0.00	0.00	0.00	0.00	1.51	1.26
Te	27	0.00	0.00	0.00	0.00	0.00	0.00	NaN	NaN
Tl	27	0.00	0.00	0.00	0.00	0.00	0.00	1.59	1.38
Tm	27	0.00	0.00	0.00	0.00	0.00	0.02	3.48	10.99
V	27	0.02	0.02	0.01	0.01	0.00	0.09	1.24	0.81
Yb	27	0.00	0.00	0.00	0.00	0.00	0.00	NaN	NaN
Zn	27	7.54	2.88	6.95	3.02	3.45	14.77	0.82	0.37

(b) Descriptive statistics for the content of elements in the right lung tissue for the studied population (smoker + nonsmoker).

Group	n_Obs	Mean	SD	Median	MAD	Min	Max	Skewness	Kurtosis
Al	27	8.51	9.13	5.49	5.04	0	34.18	1.78	2.91
As	27	0	0	0	0	0	0.01	3.45	10.68
B	27	0	0	0	0	0	0	NaN	NaN
Ba	27	0	0	0	0	0	0	NaN	NaN
Be	27	0	0	0	0	0	0	NaN	NaN
Ca	27	88.43	91.38	65.36	27.7	0	493.46	3.56	15.5
Cd	27	0.13	0.16	0.07	0.08	0	0.69	2.11	4.96
Ce	27	0.01	0.01	0.01	0.01	0	0.05	1.7	2.4
Co	27	0	0	0	0	0	0.01	0.69	−0.20
Cr	27	0.07	0.07	0.05	0.02	0.01	0.32	2.9	8.91
Cs	27	0	0	0	0	0	0.01	0.7	−0.57
Cu	27	0.7	1.03	0.32	0.37	0	4.28	2.19	4.82
Dy	27	0	0	0	0	0	0	1.81	2.58
Er	27	0	0	0	0	0	0	3.14	11.44
Eu	27	0	0	0	0	0	0	3.45	10.67
Fe	27	170.6	76.58	178.29	92.47	15.95	323.71	0.21	−0.39
Gd	27	0	0	0	0	0	0	3.45	10.7
Ho	27	0	0	0	0	0	0	NaN	NaN
K	27	1549.49	418.14	1606.77	382.83	580.88	2264.63	−0.75	0.06
La	27	0	0.02	0	0	0	0.09	4.77	23.72
Lu	27	0	0	0	0	0	0	NaN	NaN
Mg	27	63.44	11.61	63.83	10.5	40.82	90.42	0.12	0.2
Mn	27	0.22	0.16	0.21	0.14	0	0.74	1.21	2.87

Na	27	1341.1	372.07	1315.33	411.97	631.09	2391.14	0.64	1.11
Nd	27	0	0	0	0	0	0.02	2.81	8.84
Ni	27	0.03	0.09	0	0.01	0	0.46	4.97	25.3
P	27	1006.41	233.67	1002.22	202.46	574.58	1451.19	0.05	−0.45
Pb	27	0.05	0.13	0.01	0.02	0	0.52	3.32	10.3
Pr	27	0	0	0	0	0	0.02	5.1	26.32
Rb	27	1.66	0.56	1.73	0.63	0.42	2.48	−0.57	−0.23
Sb	27	0	0.01	0	0	0	0.03	3.66	12.81
Se	27	0.16	0.05	0.16	0.03	0.08	0.3	0.86	1.68
Sm	27	0	0	0	0	0	0	NaN	NaN
Sr	27	0.4	0.38	0.24	0.16	0.03	1.53	1.66	2.4
Tb	27	0	0	0	0	0	0	2.47	7.79
Te	27	0	0	0	0	0	0	NaN	NaN
Tl	27	0	0	0	0	0	0	1.32	1.04
Tm	27	0	0	0	0	0	0	NaN	NaN
V	27	0.03	0.03	0.02	0.03	0	0.11	1.04	0.86
Yb	27	0	0	0	0	0	0	NaN	NaN
Zn	27	7.83	2.06	7.61	2.17	4.98	13.37	0.81	0.54

(c) Descriptive statistics of the content of elements in the left lung tissue for the studied population (smoker + non-smoker).

Group	n_Obs	Mean	SD	Median	MAD	Min	Max	Skewness	Kurtosis
Al	28	14.01	20.33	4.58	5.25	0	81.62	2.21	4.66
As	28	0	0	0	0	0	0	NaN	NaN
B	28	0	0	0	0	0	0	NaN	NaN
Ba	28	0.01	0.04	0	0	0	0.19	3.53	13.03
Be	28	0	0	0	0	0	0	5.29	28
Ca	28	168.81	390.85	82.84	45.94	0	2121.16	4.96	25.45
Cd	28	0.11	0.12	0.07	0.08	0.01	0.45	1.7	2.6
Ce	28	0.02	0.03	0.01	0.01	0	0.12	2.07	4.37
Co	28	0	0	0	0	0	0.02	1.32	1.39
Cr	28	0.1	0.13	0.06	0.06	0	0.63	2.68	8.54
Cs	28	0	0	0	0	0	0.02	1.81	3.33
Cu	28	0.69	1.04	0.34	0.33	0	4	2.34	4.69
Dy	28	0	0	0	0	0	0	1.83	2.77
Er	28	0	0	0	0	0	0.01	3.09	9.13
Eu	28	0	0	0	0	0	0	4.41	20.19
Fe	28	162.55	72.77	155.6	97.65	30.1	282.94	0.06	-1.33
Gd	28	0	0	0	0	0	0.01	4.92	25.05
Ho	28	0	0	0	0	0	0	NaN	NaN
K	28	1539.29	454.46	1612.07	447.08	753.47	2301.7	-0.3	-0.97
La	28	0.02	0.09	0	0	0	0.49	5.17	27.07
Lu	28	0	0	0	0	0	0	NaN	NaN
Mg	28	67.23	20.36	68.84	23.89	32.74	126.21	0.79	1.2
Mn	28	0.28	0.29	0.22	0.15	0	1.61	3.94	18.48
Na	28	1394.69	354.1	1365.63	441.7	821.29	2093.65	0.35	-0.77

Nd	28	0	0.01	0	0	0	0.04	2.93	9.85
Ni	28	0.03	0.05	0.01	0.01	0	0.19	2.43	5.2
P	28	1084.93	446.66	1024.88	348.83	616.38	2694.08	1.88	5.12
Pb	28	0.04	0.04	0.02	0.02	0	0.15	1.47	1.23
Pr	28	0	0	0	0	0	0.01	4.25	19.93
Rb	28	1.68	0.63	1.82	0.73	0.63	2.76	-0.21	-1.07
Sb	28	0	0.01	0	0	0	0.03	3.37	11.25
Se	28	0.19	0.08	0.17	0.05	0.08	0.52	2.43	8.4
Sm	28	0	0	0	0	0	0	4.52	21.86
Sr	28	0.45	0.32	0.37	0.31	0.1	1.25	1.09	0.65
Tb	28	0	0	0	0	0	0	2.07	3.04
Te	28	0	0	0	0	0	0	NaN	NaN
Tl	28	0	0	0	0	0	0	1.88	2.88
Tm	28	0	0	0	0	0	0	5.29	28
V	28	0.04	0.04	0.03	0.04	0	0.17	1.47	2.8
Yb	28	0	0	0	0	0	0	NaN	NaN
Zn	28	8.63	2.85	8.21	2.59	3.71	16.35	0.75	0.57

(d) Descriptive statistics of element contents in liver tissue (μg/g w.w.) for the study population.

**Group**	**n_Obs**	**Mean**	**SD**	**Median**	**MAD**	**Min**	**Max**	**Skewness**	**Kurtosis**
Al	26	0.59	1.94	0	0	0	9.61	4.42	20.55
As	26	0	0	0	0	0	0.01	1.85	1.85
B	26	0	0	0	0	0	0	NaN	NaN
Ba	26	0	0	0	0	0	0	NaN	NaN
Be	26	0	0	0	0	0	0	NaN	NaN
Ca	26	41.8	24.81	40.46	22.55	0	87.59	-0.19	-0.43
Cd	26	0.43	0.43	0.22	0.2	0.06	1.53	1.39	0.97
Ce	26	0.01	0.02	0.01	0.01	0	0.1	3.89	17.13
Co	26	0.01	0.01	0.01	0.01	0	0.03	1.12	1.82
Cr	26	0.02	0.02	0.02	0.02	0	0.07	1.35	1.3
Cs	26	0.01	0.01	0.01	0	0	0.02	0.48	-0.38
Cu	26	3.86	3.57	2.77	2.71	0	15.87	1.65	3.87
Dy	26	0	0	0	0	0	0	NaN	NaN
Er	26	0	0	0	0	0	0	NaN	NaN
Eu	26	0	0	0	0	0	0	NaN	NaN
Fe	26	177.12	143.44	138.97	76.66	39.95	727.46	2.49	8.08
Gd	26	0.01	0.05	0	0	0	0.26	5.07	25.82
Ho	26	0	0	0	0	0	0	NaN	NaN
K	26	2367.12	541.72	2319.58	560.9	1362.33	3544.81	0.13	-0.23
La	26	0	0.01	0	0	0	0.06	4.13	18.67
Lu	26	0	0	0	0	0	0	NaN	NaN
Mg	26	147.14	34.27	137.96	32.36	101.12	217.33	0.74	-0.49
Mn	26	3.33	1.19	3.17	0.9	1.12	6.56	0.53	1.13
Na	26	1338.62	478.94	1257.93	347.45	758.94	2813.45	1.58	3.03
Nd	26	0	0	0	0	0	0.01	1.65	2.21
Ni	26	0.05	0.12	0	0	0	0.52	3.15	9.94
P	26	2482.32	542.41	2347.2	413.36	1887.78	3967.54	1.44	1.76

Pb	26	0.13	0.16	0.08	0.05	0	0.79	3.21	12.57
Pr	26	0	0	0	0	0	0	2.29	5.1
Rb	26	3.42	1.53	3.46	1.39	1.27	6.98	0.69	0.36
Sb	26	0	0	0	0	0	0	NaN	NaN
Se	26	0.52	0.35	0.43	0.15	0.23	2.09	3.84	17.28
Sm	26	0	0	0	0	0	0	5.1	26
Sr	26	0.14	0.16	0.1	0.1	0	0.75	2.64	9.02
Tb	26	0	0	0	0	0	0	2.62	7.53
Te	26	0	0	0	0	0	0	NaN	NaN
Tl	26	0	0	0	0	0	0	1.93	2.39
Tm	26	0	0	0	0	0	0.01	4.47	20.7
V	26	0.01	0.01	0	0	0	0.04	0.66	-1.23
Yb	26	0	0	0	0	0	0	NaN	NaN
Zn	26	48.02	34.31	35.03	12.13	13.2	178.29	2.47	7.64

(e) Descriptive statistics for the study population based on measurements of element levels in whole brain tissues.

Group	n_Obs	Mean	SD	Median	MAD	Min	Max	Skewness	Kurtosis
Al	288	0.34	1.44	0	0	0	18.01	8.21	85.89
As	288	0	0.01	0	0	0	0.06	5.33	31.24
B	288	0	0	0	0	0	0	NaN	NaN
Ba	288	0.01	0.06	0	0	0	0.98	14.91	238.15
Be	288	0	0	0	0	0	0	NaN	NaN
Ca	288	78.19	211.33	43.8	36.51	0	3176.19	11.63	163.38
Cd	288	0.01	0.02	0.01	0.01	0	0.25	5.23	40.1
Ce	288	0	0	0	0	0	0.04	6.44	52.8
Co	288	0	0	0	0	0	0.03	11.09	131.95
Cr	288	0.04	0.12	0.02	0.03	0	1.8	12.14	171.96
Cs	288	0	0.01	0	0	0	0.05	3.41	17.3
Cu	288	3.88	4.84	2.38	3.53	0	29.63	2.46	8.28
Dy	288	0	0	0	0	0	0	7.89	63.08
Er	288	0	0	0	0	0	0	16.97	288
Eu	288	0	0	0	0	0	0	16.97	288
Fe	288	57.17	44.72	41.28	18.99	16.04	372.05	3.39	16.63
Gd	288	0	0	0	0	0	0.05	11.17	142.1
Ho	288	0	0	0	0	0	0	NaN	NaN
K	288	2762.89	939.93	2648.66	557.7	733.39	10335.27	4.04	26.14
La	288	0	0.01	0	0	0	0.19	11.44	137.34
Lu	288	0	0	0	0	0	0	NaN	NaN
Mg	288	125.16	42.69	116.75	21.19	31.58	476.33	4.3	27.4
Mn	288	0.75	0.48	0.64	0.33	0	4.22	2.46	11.52
Na	288	1978.97	774.84	1835.91	414.06	756.37	9811.63	4.87	39.67

Nd	288	0	0	0	0	0	0.01	5.6	32.79
Ni	288	0.04	0.17	0	0	0	2.08	8.04	79.76
P	288	2968.44	1097.25	2647.45	615.06	797.93	9806.65	2.42	9.35
Pb	288	0.17	0.45	0.01	0.02	0	5.47	7.07	69.93
Pr	288	0	0	0	0	0	0	7.92	64.87
Rb	288	2.09	0.99	1.98	0.72	0.58	9	2.89	15.77
Sb	288	0	0.01	0	0	0	0.23	16.97	288
Se	288	0.21	0.09	0.19	0.05	0.09	0.9	3.14	16.69
Sm	288	0	0	0	0	0	0	NaN	NaN
Sr	288	0.12	0.25	0.07	0.1	0	3.01	7.41	70.42
Tb	288	0	0	0	0	0	0.01	2.29	4.62
Te	288	0	0	0	0	0	0	NaN	NaN
Tl	288	0	0	0	0	0	0.01	4.76	28.1
Tm	288	0	0	0	0	0	0.04	7.49	69.5
V	288	0.04	0.07	0.02	0.03	0	0.92	7.27	82.78
Yb	288	0	0	0	0	0	0	NaN	NaN
Zn	288	9.86	4.92	9.4	3.07	0	43.21	2.6	12.44

(f) Descriptive statistics of the content of chemical elements in μg/g w.w. in brain area A for the studied population.

Group	n_Obs	Mean	SD	Median	MAD	Min	Max	Skewness	Kurtosis
Al	28	0	0	0	0	0	0	NaN	NaN
As	28	0	0	0	0	0	0.02	5.29	28
B	28	0	0	0	0	0	0	NaN	NaN
Ba	28	0	0.02	0	0	0	0.12	5.29	28
Be	28	0	0	0	0	0	0	NaN	NaN
Ca	28	93.04	132.6	61.42	58.09	0	626.37	3.04	10.25
Cd	28	0.01	0.01	0.01	0.01	0	0.05	1.31	1.65
Ce	28	0	0	0	0	0	0.01	2.56	5.71
Co	28	0	0	0	0	0	0	NaN	NaN
Cr	28	0.04	0.04	0.03	0.02	0	0.16	1.85	4
Cs	28	0	0	0	0	0	0.01	5.29	28
Cu	28	3.95	4.16	2.57	3.73	0	14.19	1.1	0.3
Dy	28	0	0	0	0	0	0	NaN	NaN
Er	28	0	0	0	0	0	0	NaN	NaN
Eu	28	0	0	0	0	0	0	NaN	NaN
Fe	28	33.42	9.42	31.94	7.08	19.21	59.65	1.21	2.25
Gd	28	0	0	0	0	0	0	5.29	28
Ho	28	0	0	0	0	0	0	NaN	NaN
K	28	2317.68	341.41	2301.7	302.39	1604.98	2857.74	−0.19	−0.55

La	28	0.01	0.04	0	0	0	0.19	5.28	27.94
Lu	28	0	0	0	0	0	0	NaN	NaN
Mg	28	107.8	20.47	106.74	14.57	78.29	183.02	1.8	5.8
Mn	28	0.42	0.21	0.42	0.18	0	0.8	0.02	−0.61
Na	28	2232.19	458.47	2117.82	304.33	1683.18	3417.9	1.4	1.38
Nd	28	0	0	0	0	0	0	NaN	NaN
Ni	28	0.01	0.01	0	0	0	0.06	2.61	6.56
P	28	2332.93	479.46	2242.81	272.41	1576.74	4272.3	2.53	9.66
Pb	28	0.14	0.31	0	0	0	1.28	3.06	9.05
Pr	28	0	0	0	0	0	0	NaN	NaN
Rb	28	1.46	0.44	1.45	0.58	0.66	2.26	0.06	−0.76
Sb	28	0	0	0	0	0	0	NaN	NaN
Se	28	0.19	0.05	0.19	0.05	0.13	0.38	1.79	5.52
Sm	28	0	0	0	0	0	0	NaN	NaN
Sr	28	0.16	0.32	0.06	0.09	0	1.63	4.04	18.51
Tb	28	0	0	0	0	0	0	4.39	19.97
Te	28	0	0	0	0	0	0	NaN	NaN
Tl	28	0	0	0	0	0	0	3.52	11.18
Tm	28	0	0	0	0	0	0.01	5.29	28
V	28	0.04	0.04	0.03	0.04	0	0.15	1.11	0.29
Yb	28	0	0	0	0	0	0	NaN	NaN
Zn	28	9.4	2.96	9.32	1.93	1.63	15.72	−0.29	1.04

(g) Descriptive statistics of the chemical elements content in μg/g w.w. in brain area B for the studied population.

Group	n_Obs	Mean	SD	Median	MAD	Min	Max	Skewness	Kurtosis
Al	26	0.12	0.42	0	0	0	1.59	3.38	10.2
As	26	0	0.01	0	0	0	0.06	4.44	20.5
B	26	0	0	0	0	0	0	NaN	NaN
Ba	26	0.01	0.03	0	0	0	0.14	5.1	26
Be	26	0	0	0	0	0	0	NaN	NaN
Ca	26	36.28	37.27	29.56	34.5	0	154.47	1.46	2.75
Cd	26	0.01	0.01	0	0	0	0.04	1.13	0.5
Ce	26	0	0	0	0	0	0	NaN	NaN
Co	26	0	0.01	0	0	0	0.03	5.1	26
Cr	26	0.04	0.04	0.03	0.04	0	0.14	0.93	0.75
Cs	26	0	0	0	0	0	0.02	2.85	8.62
Cu	26	1.87	2.63	0.9	1.34	0	10.47	1.8	3.42
Dy	26	0	0	0	0	0	0	NaN	NaN
Er	26	0	0	0	0	0	0	NaN	NaN
Eu	26	0	0	0	0	0	0	NaN	NaN

Fe	26	53.01	11.13	53.15	8.45	28.91	74.66	−0.52	0.26
Gd	26	0	0	0	0	0	0	NaN	NaN
Ho	26	0	0	0	0	0	0	NaN	NaN
K	26	2380.99	452.26	2294.85	271.43	1706.05	3515.22	0.79	0.3
La	26	0	0	0	0	0	0	NaN	NaN
Lu	26	0	0	0	0	0	0	NaN	NaN
Mg	26	106.62	13.97	106.24	13.96	84.9	138.7	0.42	−0.28
Mn	26	0.53	0.2	0.48	0.16	0.23	1.15	1.39	2.71
Na	26	2190.87	311.85	2128.63	374.6	1639.87	2872.3	0.49	−0.31
Nd	26	0	0	0	0	0	0	NaN	NaN
Ni	26	0.02	0.06	0	0	0	0.27	3.61	12.96
P	26	2542.93	372.72	2417.23	299.39	1947.2	3275.25	0.59	−0.84
Pb	26	0.06	0.12	0	0	0	0.52	3.04	9.75
Pr	26	0	0	0	0	0	0	NaN	NaN
Rb	26	1.64	0.55	1.53	0.54	0.66	3.11	0.69	0.73
Sb	26	0	0	0	0	0	0	NaN	NaN
Se	26	0.2	0.06	0.19	0.05	0.14	0.43	2.32	7.47
Sm	26	0	0	0	0	0	0	NaN	NaN
Sr	26	0.05	0.06	0.04	0.06	0	0.24	1.29	1.71
Tb	26	0	0	0	0	0	0.01	1.64	1.76
Te	26	0	0	0	0	0	0	NaN	NaN
Tl	26	0	0	0	0	0	0	1.31	0.11
Tm	26	0	0	0	0	0	0	NaN	NaN
V	26	0.05	0.07	0.02	0.03	0	0.29	1.95	4.17
Yb	26	0	0	0	0	0	0	NaN	NaN
Zn	26	7.97	2.84	7.27	2.14	2.73	14.46	0.6	0.19

(h) Descriptive statistics of element content in brain area C for the studied population.

Group	n_Obs	Mean	SD	Median	MAD	Min	Max	Skewness	Kurtosis
Al	27	0.36	1.57	0	0	0	8.07	4.94	24.96
As	27	0	0.01	0	0	0	0.05	5.2	27
B	27	0	0	0	0	0	0	NaN	NaN
Ba	27	0	0	0	0	0	0	NaN	NaN
Be	27	0	0	0	0	0	0	NaN	NaN
Ca	27	53.93	45.7	53.05	43.76	0	169.81	0.93	0.44
Cd	27	0.01	0.01	0.01	0.01	0	0.05	1.17	1.02
Ce	27	0	0	0	0	0	0.02	4.05	17.8
Co	27	0	0	0	0	0	0	NaN	NaN
Cr	27	0.03	0.04	0.02	0.03	0	0.16	1.9	5.28
Cs	27	0	0	0	0	0	0.02	2.75	8.31

Cu	27	4.81	6.73	2.83	4.19	0	26.45	2.09	4.39
Dy	27	0	0	0	0	0	0	5.2	27
Er	27	0	0	0	0	0	0	NaN	NaN
Eu	27	0	0	0	0	0	0	NaN	NaN
Fe	27	52.42	30.72	43.79	13.43	28.96	191.6	3.8	17.14
Gd	27	0	0	0	0	0	0.01	5.2	27
Ho	27	0	0	0	0	0	0	NaN	NaN
K	27	2545.19	1203.17	2269.17	406.92	1172.49	7699.97	3.26	13.28
La	27	0	0.01	0	0	0	0.03	5.2	27
Lu	27	0	0	0	0	0	0	NaN	NaN
Mg	27	113.47	47.15	102.94	10.58	58.47	333.71	4.16	19.83
Mn	27	0.58	0.46	0.47	0.18	0.21	2.63	3.78	16.72
Na	27	2231.92	582.32	2228.94	311.17	963.43	4514.26	2.1	9.33
Nd	27	0	0	0	0	0	0	5.2	27
Ni	27	0.02	0.09	0	0	0	0.44	4.75	23.46
P	27	2699.54	1171.96	2485.88	341.28	1368.92	7943.1	3.68	16.28
Pb	27	0.17	0.3	0	0	0	1.17	2.08	4.03
Pr	27	0	0	0	0	0	0	5.2	27
Rb	27	1.79	1.21	1.52	0.57	0.75	7	3.27	13.3
Sb	27	0.01	0.04	0	0	0	0.23	5.2	27
Se	27	0.21	0.1	0.19	0.04	0.09	0.63	3.26	13.94
Sm	27	0	0	0	0	0	0	NaN	NaN
Sr	27	0.1	0.11	0.08	0.11	0	0.43	1.6	3.1
Tb	27	0	0	0	0	0	0.01	2.47	6.05
Te	27	0	0	0	0	0	0	NaN	NaN
Tl	27	0	0	0	0	0	0	2.31	4.75
Tm	27	0	0	0	0	0	0.02	4.47	20.76
V	27	0.03	0.04	0	0	0	0.13	1.54	1.75
Yb	27	0	0	0	0	0	0	NaN	NaN
Zn	27	9.22	5.95	7.88	3.31	0	29.28	1.8	4.56

(i) Descriptive statistics of the chemical elements content in μg/g w.w. in the brain area D for the studied population.

Group	n_Obs	Mean	SD	Median	MAD	Min	Max	Skewness	Kurtosis
Al	26	0.21	0.62	0	0	0	2.62	3.16	9.94
As	26	0	0	0	0	0	0.01	5.1	26
B	26	0	0	0	0	0	0	NaN	NaN
Ba	26	0.01	0.04	0	0	0	0.18	3.81	14.52
Be	26	0	0	0	0	0	0	NaN	NaN
Ca	26	69.34	103.92	47.22	27.4	0	552.62	4.31	20.46
Cd	26	0.01	0.01	0	0	0	0.03	1.4	1.51

Ce	26	0	0	0	0	0	0	3.4	10.43
Co	26	0	0	0	0	0	0	NaN	NaN
Cr	26	0.04	0.04	0.02	0.03	0	0.15	1.49	1.6
Cs	26	0	0	0	0	0	0.01	1.3	0.32
Cu	26	3.33	3.86	2.32	3.44	0	15.31	1.65	2.87
Dy	26	0	0	0	0	0	0	NaN	NaN
Er	26	0	0	0	0	0	0	NaN	NaN
Eu	26	0	0	0	0	0	0	NaN	NaN
Fe	26	32.7	11.51	29.14	7.24	20.53	62.08	1.29	0.74
Gd	26	0	0	0	0	0	0	5.1	26
Ho	26	0	0	0	0	0	0	NaN	NaN
K	26	2949.39	532.13	2881.97	473.78	1969.79	4124.31	0.43	0.06
La	26	0	0	0	0	0	0	NaN	NaN
Lu	26	0	0	0	0	0	0	NaN	NaN
Mg	26	121.99	27.41	114.17	16.56	84.01	210.94	1.6	3.37
Mn	26	0.47	0.18	0.46	0.13	0.17	0.89	0.62	0.83
Na	26	1902.29	476.09	1823.92	260.71	1376.15	3814.92	2.74	10.22
Nd	26	0	0	0	0	0	0	NaN	NaN
Ni	26	0.02	0.08	0	0	0	0.4	4.7	22.9
P	26	2467.77	335.33	2372.23	292.85	1960.79	3296.12	0.99	0.72
Pb	26	0.12	0.25	0	0	0	1.02	2.76	7.75
Pr	26	0	0	0	0	0	0	5.1	26
Rb	26	2.11	0.64	2.13	0.85	1.15	3.45	0.28	−0.96
Sb	26	0	0	0	0	0	0	NaN	NaN
Se	26	0.2	0.05	0.2	0.05	0.13	0.33	0.71	0.18
Sm	26	0	0	0	0	0	0	NaN	NaN
Sr	26	0.15	0.34	0.07	0.1	0	1.76	4.72	23.33
Tb	26	0	0	0	0	0	0	1.56	0.88
Te	26	0	0	0	0	0	0	NaN	NaN
Tl	26	0	0	0	0	0	0.01	2.55	7.13
Tm	26	0	0.01	0	0	0	0.04	4.63	22.14
V	26	0.04	0.05	0.02	0.03	0	0.19	1.79	2.37
Yb	26	0	0	0	0	0	0	NaN	NaN
Zn	26	10.45	2.22	10.27	1.76	6.51	15.89	0.63	0.28

(j) Descriptive statistics of the chemical elements content in µg/g w.w. in the brain region E for the studied population.

Group	n_Obs	Mean	SD	Median	MAD	Min	Max	Skewness	Kurtosis
Al	25	0.21	0.53	0	0	0	1.89	2.38	4.58
As	25	0	0	0	0	0	0	NaN	NaN
B	25	0	0	0	0	0	0	NaN	NaN

Ba	25	0	0.01	0	0	0	0.05	5	25
Be	25	0	0	0	0	0	0	NaN	NaN
Ca	25	78.66	138.93	40.45	39.97	0	521.88	2.66	6.35
Cd	25	0.01	0.02	0.01	0.01	0	0.07	2.05	5.15
Ce	25	0	0.01	0	0	0	0.02	3.14	10.12
Co	25	0	0	0	0	0	0	NaN	NaN
Cr	25	0.02	0.02	0.01	0.01	0	0.09	1.42	1.23
Cs	25	0	0	0	0	0	0.01	1.13	0.01
Cu	25	3.7	5.91	1.73	2.56	0	27.7	3.18	11.58
Dy	25	0	0	0	0	0	0	5	25
Er	25	0	0	0	0	0	0	NaN	NaN
Eu	25	0	0	0	0	0	0	5	25
Fe	25	32.05	18.06	28.4	5.58	16.04	114.55	4.26	19.96
Gd	25	0	0	0	0	0	0.01	5	25
Ho	25	0	0	0	0	0	0	NaN	NaN
K	25	2730.38	1457.57	2579.72	388.83	1388.64	9433.92	4.32	20.61
La	25	0	0	0	0	0	0	NaN	NaN
Lu	25	0	0	0	0	0	0	NaN	NaN
Mg	25	126.59	75.13	111.65	13.87	77.48	476.33	4.54	21.75
Mn	25	0.62	0.37	0.53	0.17	0.11	2.15	3.2	13.48
Na	25	2055.83	1658.28	1670.93	294.35	1302.28	9811.63	4.62	22.21
Nd	25	0	0	0	0	0	0.01	3.65	13.09
Ni	25	0.03	0.07	0	0	0	0.24	2.96	7.9
P	25	2633.43	1540.57	2339.16	227.97	1667.48	9806.65	4.55	21.78
Pb	25	0.14	0.2	0.02	0.03	0	0.64	1.47	1.08
Pr	25	0	0	0	0	0	0	NaN	NaN
Rb	25	2.02	0.83	2	0.62	0.71	4.71	1.25	3.52
Sb	25	0	0	0	0	0	0	NaN	NaN
Se	25	0.2	0.15	0.17	0.03	0.11	0.9	4.3	19.94
Sm	25	0	0	0	0	0	0	NaN	NaN
Sr	25	0.07	0.12	0.04	0.06	0	0.53	2.95	9.72
Tb	25	0	0	0	0	0	0	3.96	16.06
Te	25	0	0	0	0	0	0	NaN	NaN
Tl	25	0	0	0	0	0	0.01	4.9	24.23
Tm	25	0	0	0	0	0	0.01	3.3	9.69
V	25	0.04	0.05	0.02	0.04	0	0.21	2.35	5.45
Yb	25	0	0	0	0	0	0	NaN	NaN
Zn	25	11.13	2.62	10.54	1.65	6.92	20.12	1.8	5.06

(k) Descriptive statistics of the chemical elements content in μg/g w.w. in the brain area F for the studied population.

Group	n_Obs	Mean	SD	Median	MAD	Min	Max	Skewness	Kurtosis
Al	26	0.55	1.63	0	0	0	7.48	3.66	14.1
As	26	0	0.01	0	0	0	0.03	4.24	19.08
B	26	0	0	0	0	0	0	NaN	NaN
Ba	26	0	0	0	0	0	0	NaN	NaN
Be	26	0	0	0	0	0	0	NaN	NaN
Ca	26	58.31	36.95	59.22	32.04	0	174.79	0.98	2.87
Cd	26	0.02	0.02	0.01	0.02	0	0.08	1.94	3.88
Ce	26	0	0	0	0	0	0.02	2.68	6.26
Co	26	0	0	0	0	0	0	NaN	NaN
Cr	26	0.03	0.05	0.02	0.02	0	0.24	3.83	16.88
Cs	26	0	0.01	0	0	0	0.02	1.27	0.98
Cu	26	4.99	4.58	4.16	2.06	0	22.28	2.43	7.68
Dy	26	0	0	0	0	0	0	NaN	NaN
Er	26	0	0	0	0	0	0	NaN	NaN
Eu	26	0	0	0	0	0	0	NaN	NaN
Fe	26	110.6	36.35	100.36	26.33	70.48	218.29	1.52	2.29
Gd	26	0	0	0	0	0	0.02	4.52	21.21
Ho	26	0	0	0	0	0	0	NaN	NaN
K	26	2792.23	639.57	2759.52	570.45	1863.93	4446.17	1.2	1.71
La	26	0	0	0	0	0	0	NaN	NaN
Lu	26	0	0	0	0	0	0	NaN	NaN
Mg	26	124.91	22.05	122.35	12.84	95.59	188.38	1.64	3.16
Mn	26	1.12	0.28	1.11	0.14	0.47	1.68	−0.06	0.11
Na	26	2101.87	777.11	1823.83	292.44	1493.64	4961.33	2.52	6.98
Nd	26	0	0	0	0	0	0.01	2.77	6.59
Ni	26	0.03	0.06	0	0	0	0.21	2.18	3.78
P	26	2722.8	524.42	2707.69	230.44	1997.76	4246.82	1.51	2.87
Pb	26	0.16	0.3	0.08	0.12	0	1.48	3.56	14.83
Pr	26	0	0	0	0	0	0	5.1	26
Rb	26	2.31	0.92	2.2	0.74	1.06	5.5	1.84	4.99
Sb	26	0	0	0	0	0	0	NaN	NaN
Se	26	0.25	0.07	0.24	0.08	0.14	0.47	1	1.57
Sm	26	0	0	0	0	0	0	NaN	NaN
Sr	26	0.08	0.06	0.07	0.07	0	0.22	0.42	−0.61
Tb	26	0	0	0	0	0	0	4.37	19.83
Te	26	0	0	0	0	0	0	NaN	NaN
Tl	26	0	0	0	0	0	0	2.34	4.38
Tm	26	0	0	0	0	0	0.02	3.5	11.46

V	26	0.03	0.05	0.01	0.02	0	0.23	3.09	10.56
Yb	26	0	0	0	0	0	0	NaN	NaN
Zn	26	11.9	4.78	10.81	2.87	6.57	27.75	2.08	4.85

(l) Descriptive statistics of the chemical elements content in μg/g w.w. in the brain area G for the studied population.

Group	n_Obs	Mean	SD	Median	MAD	Min	Max	Skewness	Kurtosis
Al	27	0.15	0.38	0	0	0	1.25	2.24	3.55
As	27	0	0.01	0	0	0	0.03	2.93	7.89
B	27	0	0	0	0	0	0	NaN	NaN
Ba	27	0.01	0.02	0	0	0	0.1	3.54	11.61
Be	27	0	0	0	0	0	0	NaN	NaN
Ca	27	77.72	94.76	51.52	54.2	0	357.32	1.83	2.87
Cd	27	0.01	0.01	0.01	0.01	0	0.05	1.45	1.95
Ce	27	0	0	0	0	0	0.01	4.73	23.13
Co	27	0	0	0	0	0	0	NaN	NaN
Cr	27	0.04	0.04	0.03	0.02	0	0.16	1.92	4.49
Cs	27	0	0.01	0	0	0	0.03	2.33	6.23
Cu	27	4.77	4.39	3.22	4.59	0	13.43	0.69	−0.80
Dy	27	0	0	0	0	0	0	5.2	27
Er	27	0	0	0	0	0	0	NaN	NaN
Eu	27	0	0	0	0	0	0	NaN	NaN
Fe	27	43.27	11.23	43.04	12.33	24.47	70.96	0.54	0.3
Gd	27	0	0	0	0	0	0.02	5.2	27
Ho	27	0	0	0	0	0	0	NaN	NaN
K	27	2849.26	580.16	2816.76	382.39	1690.05	4699.27	0.96	3.44
La	27	0	0	0	0	0	0	NaN	NaN
Lu	27	0	0	0	0	0	0	NaN	NaN
Mg	27	144.1	31.36	137.12	19.9	83.71	247.91	1.58	4.35
Mn	27	1.01	0.39	0.9	0.33	0.29	1.78	0.45	−0.43
Na	27	1547.65	315.5	1475.72	231.73	1145.61	2633.02	2.05	5.27
Nd	27	0	0	0	0	0	0.01	5.2	27
Ni	27	0.1	0.4	0	0	0	2.08	5.05	25.88
P	27	4393.59	851.05	4310.02	449.81	1868.82	6786.69	−0.15	3.97
Pb	27	0.21	0.4	0.04	0.06	0	1.7	2.73	7.83
Pr	27	0	0	0	0	0	0	NaN	NaN
Rb	27	2.18	0.82	2.1	0.71	0.89	4.35	1.01	1.6
Sb	27	0	0	0	0	0	0	NaN	NaN
Se	27	0.18	0.08	0.17	0.06	0.1	0.4	1.75	3.15
Sm	27	0	0	0	0	0	0	NaN	NaN
Sr	27	0.15	0.23	0.1	0.15	0	1.15	3.32	13.32

Tb	27	0	0	0	0	0	0	1.55	1
Te	27	0	0	0	0	0	0	NaN	NaN
Tl	27	0	0	0	0	0	0	2.04	3.74
Tm	27	0	0	0	0	0	0.02	5.2	27
V	27	0.04	0.03	0.03	0.03	0	0.13	1.18	1.92
Yb	27	0	0	0	0	0	0	NaN	NaN
Zn	27	9.14	6.02	8.11	2.12	3.92	34.65	3.26	12.75

(m) Descriptive statistics of the chemical elements content in μg/g w.w. in the brain region H for the studied population.

Group	n_Obs	Mean	SD	Median	MAD	Min	Max	Skewness	Kurtosis
Al	24	0.51	1.73	0	0	0	8.12	4.12	18.01
As	24	0	0.01	0	0	0	0.06	3.52	12.81
B	24	0	0	0	0	0	0	NaN	NaN
Ba	24	0.04	0.2	0	0	0	0.98	4.9	24
Be	24	0	0	0	0	0	0	NaN	NaN
Ca	24	222.88	662.07	37.95	25.75	0	3176.19	4.28	19.06
Cd	24	0.01	0.01	0.01	0.02	0	0.04	0.81	0.25
Ce	24	0	0	0	0	0	0.01	4.77	23.07
Co	24	0	0	0	0	0	0	4.9	24
Cr	24	0.03	0.04	0.02	0.03	0	0.11	0.84	−0.49
Cs	24	0	0.01	0	0	0	0.03	2.05	3.82
Cu	24	5.08	8.04	2.06	3.05	0	29.63	2.4	5.33
Dy	24	0	0	0	0	0	0	3.3	9.87
Er	24	0	0	0	0	0	0	4.9	24
Eu	24	0	0	0	0	0	0	NaN	NaN
Fe	24	42.45	13.44	39.84	11.73	19.63	78.22	0.98	1.65
Gd	24	0	0	0	0	0	0	NaN	NaN
Ho	24	0	0	0	0	0	0	NaN	NaN
K	24	2829.08	458.6	2896.88	436.51	1889.01	3566.96	−0.30	−0.58
La	24	0	0.01	0	0	0	0.06	4.9	24
Lu	24	0	0	0	0	0	0	NaN	NaN
Mg	24	144.75	24.49	143.62	27.84	105.7	188.39	0.05	−1.04
Mn	24	0.9	0.41	0.77	0.19	0	1.99	0.76	1.62
Na	24	1522.83	259.28	1435.29	159.17	1181.48	2250.47	1.22	1.39
Nd	24	0	0	0	0	0	0.01	4.9	24
Ni	24	0.09	0.29	0	0	0	1.3	3.89	15.61
P	24	4320.83	594.87	4321.86	616.84	3321.87	5415.33	0.08	−0.58
Pb	24	0.29	0.51	0.03	0.05	0	1.7	2.03	2.99
Pr	24	0	0	0	0	0	0	NaN	NaN
Rb	24	2.26	0.76	2.37	0.7	0.81	4.2	0.44	0.81

Sb	24	0	0	0	0	0	0	NaN	NaN
Se	24	0.18	0.06	0.17	0.06	0.1	0.39	1.59	4.26
Sm	24	0	0	0	0	0	0	NaN	NaN
Sr	24	0.23	0.6	0.09	0.09	0	3.01	4.72	22.78
Tb	24	0	0	0	0	0	0	1.65	0.95
Te	24	0	0	0	0	0	0	NaN	NaN
Tl	24	0	0	0	0	0	0	3.8	14.65
Tm	24	0	0	0	0	0	0	NaN	NaN
V	24	0.03	0.04	0.02	0.04	0	0.12	0.82	−0.73
Yb	24	0	0	0	0	0	0	NaN	NaN
Zn	24	8.7	8.4	6.72	1.65	0.8	43.21	3.42	13.13

(n) Descriptive statistics of the chemical elements content in μg/g w.w. in brain area I for the studied population.

Group	n_Obs	Mean	SD	Median	MAD	Min	Max	Skewness	Kurtosis
Al	28	0.22	0.71	0	0	0	3.51	4.1	18.34
As	28	0	0.01	0	0	0	0.04	3.39	11.37
B	28	0	0	0	0	0	0	NaN	NaN
Ba	28	0	0	0	0	0	0	NaN	NaN
Be	28	0	0	0	0	0	0	NaN	NaN
Ca	28	79.62	128.98	45.59	24.91	0	635.23	3.66	14.03
Cd	28	0.02	0.03	0.01	0.02	0	0.14	2.58	8.6
Ce	28	0	0	0	0	0	0.01	2.6	5.95
Co	28	0	0	0	0	0	0	5.29	28
Cr	28	0.03	0.04	0.01	0.02	0	0.16	1.84	2.61
Cs	28	0	0.01	0	0	0	0.05	4.2	19.77
Cu	28	3.79	4.06	2.04	3.02	0	12.73	1	−0.19
Dy	28	0	0	0	0	0	0	NaN	NaN
Er	28	0	0	0	0	0	0	NaN	NaN
Eu	28	0	0	0	0	0	0	NaN	NaN
Fe	28	60.52	30.69	59.6	26.59	17.58	168.98	1.7	4.89
Gd	28	0	0.01	0	0	0	0.05	5.29	28
Ho	28	0	0	0	0	0	0	NaN	NaN
K	28	2551.8	789.76	2522.68	366.47	733.39	5737.88	2.1	10.31
La	28	0	0	0	0	0	0	NaN	NaN
Lu	28	0	0	0	0	0	0	NaN	NaN
Mg	28	125.87	43.45	123.49	18.64	31.58	314.49	2.81	14.16
Mn	28	0.82	0.27	0.78	0.25	0.28	1.48	0.42	−0.07
Na	28	2062.64	937.26	1838.77	288.93	756.37	6090.61	3.17	12.92
Nd	28	0	0	0	0	0	0.01	4.51	21.13
Ni	28	0.02	0.04	0.01	0.01	0	0.21	3.92	17.76

P	28	2784.57	513.74	2787.14	492.1	797.93	3417.46	−2.13	7.49
Pb	28	0.22	0.56	0.01	0.02	0	2.89	4.31	20.54
Pr	28	0	0	0	0	0	0	NaN	NaN
Rb	28	2.34	1.44	2.16	0.8	0.58	8.67	3.25	14.37
Sb	28	0	0	0	0	0	0	NaN	NaN
Se	28	0.24	0.07	0.22	0.05	0.15	0.45	1.61	2.34
Sm	28	0	0	0	0	0	0	NaN	NaN
Sr	28	0.12	0.18	0.07	0.07	0	0.89	3.44	13.99
Tb	28	0	0	0	0	0	0	1.79	1.76
Te	28	0	0	0	0	0	0	NaN	NaN
Tl	28	0	0	0	0	0	0	4.2	18.72
Tm	28	0	0	0	0	0	0.02	3.06	8.76
V	28	0.03	0.04	0.02	0.03	0	0.15	1.4	1.04
Yb	28	0	0	0	0	0	0	NaN	NaN
Zn	28	9.13	3.59	9.46	3.11	1.45	17	−0.02	0.12

(o). Descriptive statistics of the chemical elements content in μg/g w.w. in the brain area J for the studied population.

Group	n_Obs	Mean	SD	Median	MAD	Min	Max	Skewness	Kurtosis
Al	23	0.28	0.74	0	0	0	2.35	2.38	4.08
As	23	0	0	0	0	0	0.01	4.8	23
B	23	0	0	0	0	0	0	NaN	NaN
Ba	23	0	0	0	0	0	0	NaN	NaN
Be	23	0	0	0	0	0	0	NaN	NaN
Ca	23	35.21	33.27	29.76	39.96	0	122.09	0.85	0.46
Cd	23	0.03	0.04	0.01	0.02	0	0.18	2.48	7.37
Ce	23	0	0	0	0	0	0.01	2.65	7.06
Co	23	0	0	0	0	0	0.02	4.8	23
Cr	23	0.12	0.37	0.03	0.04	0	1.8	4.56	21.3
Cs	23	0	0.01	0	0	0	0.04	4.16	18.45
Cu	23	2.83	3.15	2.18	3.23	0	12	1.22	1.61
Dy	23	0	0	0	0	0	0	NaN	NaN
Er	23	0	0	0	0	0	0	NaN	NaN
Eu	23	0	0	0	0	0	0	NaN	NaN
Fe	23	133.56	75.59	120.34	27.71	39.66	372.05	2.42	6.02
Gd	23	0	0	0	0	0	0	NaN	NaN
Ho	23	0	0	0	0	0	0	NaN	NaN
K	23	3668.25	1718.47	3277.11	442.64	2210.06	10335.27	3.16	10.95
La	23	0	0	0	0	0	0	NaN	NaN
Lu	23	0	0	0	0	0	0	NaN	NaN

Mg	23	152.89	72.23	131.41	22.36	96.6	426.17	3.12	10.11
Mn	23	1.37	0.86	1.11	0.34	0.41	4.22	2.01	4.77
Na	23	1966.66	969.61	1642.35	551.81	1171.71	4654.14	1.95	3.49
Nd	23	0	0	0	0	0	0	4.8	23
Ni	23	0.08	0.22	0	0	0	0.96	3.31	11.46
P	23	3542.18	1487.94	3081.85	375.55	2583.47	8978.38	2.96	8.88
Pb	23	0.27	1.13	0	0	0	5.47	4.77	22.84
Pr	23	0	0	0	0	0	0	NaN	NaN
Rb	23	2.85	1.53	2.57	0.92	1.16	9	3.13	12.45
Sb	23	0	0	0	0	0	0	NaN	NaN
Se	23	0.29	0.13	0.26	0.08	0.15	0.7	2.01	4.48
Sm	23	0	0	0	0	0	0	NaN	NaN
Sr	23	0.07	0.1	0.03	0.05	0	0.43	2.57	8.32
Tb	23	0	0	0	0	0	0	2.42	4.41
Te	23	0	0	0	0	0	0	NaN	NaN
Tl	23	0	0	0	0	0	0.01	3.33	11.72
Tm	23	0	0	0	0	0	0	NaN	NaN
V	23	0.09	0.19	0.03	0.05	0	0.92	4.03	17.71
Yb	23	0	0	0	0	0	0	NaN	NaN
Zn	23	9.85	6.55	9.48	2.93	0	35.64	2.77	11.37

(p) Descriptive statistics of the chemical elements content in μg/g w.w. in the brain area K for the studied population.

Group	n_Obs	Mean	SD	Median	MAD	Min	Max	Skewness	Kurtosis
Al	28	1.07	3.46	0	0	0	18.01	4.66	23.1
As	28	0	0	0	0	0	0.02	4.15	17.67
B	28	0	0	0	0	0	0	NaN	NaN
Ba	28	0	0	0	0	0	0	NaN	NaN
Be	28	0	0	0	0	0	0	NaN	NaN
Ca	28	62.2	49.2	42.94	23.69	0	213.07	1.38	1.96
Cd	28	0.02	0.05	0.01	0.01	0	0.25	5.08	26.46
Ce	28	0	0.01	0	0	0	0.04	4.71	23.33
Co	28	0	0	0	0	0	0.02	5.22	27.44
Cr	28	0.04	0.14	0.01	0.02	0	0.73	5.16	27
Cs	28	0	0	0	0	0	0.02	1	0.17
Cu	28	3.45	3.16	2.42	3.59	0	11.1	0.82	−0.13
Dy	28	0	0	0	0	0	0	NaN	NaN
Er	28	0	0	0	0	0	0	NaN	NaN
Eu	28	0	0	0	0	0	0	NaN	NaN
Fe	28	44.88	50.74	34.56	6.09	19.35	298.71	4.98	25.63
Gd	28	0	0	0	0	0	0.01	5.29	28

Ho	28	0	0	0	0	0	0	NaN	NaN
K	28	2928.66	644.47	2926.69	458.24	1603.56	5175.48	1.24	4.82
La	28	0	0.03	0	0	0	0.14	5.29	28
Lu	28	0	0	0	0	0	0	NaN	NaN
Mg	28	114.32	24.67	114.36	18.69	79.98	211.36	2.3	8.21
Mn	28	0.52	0.23	0.5	0.15	0.13	1.25	1.17	3.14
Na	28	1906.89	401.14	1814.92	363.93	1317.86	3019.66	1.24	2.07
Nd	28	0	0	0	0	0	0	2.75	6.15
Ni	28	0.07	0.13	0	0.01	0	0.52	2.4	5.54
P	28	2429.61	488.84	2390.31	366.97	1398.38	4230.6	1.54	6.43
Pb	28	0.13	0.19	0.04	0.05	0	0.75	2.05	4.24
Pr	28	0	0	0	0	0	0	2.87	7.22
Rb	28	2.12	0.64	2.18	0.66	0.79	3.54	0	−0.21
Sb	28	0	0	0	0	0	0	NaN	NaN
Se	28	0.21	0.06	0.21	0.05	0.13	0.36	1.08	0.97
Sm	28	0	0	0	0	0	0	NaN	NaN
Sr	28	0.12	0.15	0.08	0.09	0	0.74	2.88	10.38
Tb	28	0	0	0	0	0	0	1.47	1.18
Te	28	0	0	0	0	0	0	NaN	NaN
Tl	28	0	0	0	0	0	0	4.18	17.93
Tm	28	0	0	0	0	0	0.02	5.29	28
V	28	0.02	0.02	0.02	0.03	0	0.08	0.62	−0.64
Yb	28	0	0	0	0	0	0	NaN	NaN
Zn	28	11.56	4.17	11.16	2.63	4.42	24.53	1.17	2.57

(r) Descriptive statistics of chemical element content in μg/g w.w. in the spinal cord area for the study population.

Group	n_Obs	Mean	SD	Median	MAD	Min	Max	Skewness	Kurtosis
Al	23	1.04	2.5	0	0	0	10.91	3.24	11.48
As	23	0	0.01	0	0	0	0.02	4.8	23
B	23	0	0	0	0	0	0	NaN	NaN
Ba	23	0.01	0.06	0	0	0	0.27	4.8	23
Be	23	0	0	0	0	0	0	NaN	NaN
Ca	23	190.99	540.63	57.9	64.52	0	2637.32	4.59	21.6
Cd	23	0.04	0.07	0.02	0.03	0	0.35	3.8	16.28
Ce	23	0	0	0	0	0	0.01	3.85	15.56
Co	23	0	0	0	0	0	0	NaN	NaN
Cr	23	0.13	0.23	0.05	0.04	0	1.08	3.73	15.46
Cs	23	0	0	0	0	0	0.01	2.18	3.68
Cu	23	4.12	8.76	0	0	0	32.12	2.52	5.71
Dy	23	0	0	0	0	0	0	NaN	NaN

Er	23	0	0	0	0	0	0	NaN	NaN
Eu	23	0	0	0	0	0	0	NaN	NaN
Fe	23	17.41	10.84	15.29	9.72	0	41.73	0.68	0.08
Gd	23	0	0	0	0	0	0	NaN	NaN
Ho	23	0	0	0	0	0	0	NaN	NaN
K	23	2246.26	1240.97	1965.26	187.94	1371.06	7778.45	4.37	20.17
La	23	0	0	0	0	0	0	NaN	NaN
Lu	23	0	0	0	0	0	0.01	4.8	23
Mg	23	134.38	74.92	119.43	14.37	76.65	459.17	3.99	17.7
Mn	23	0.66	0.47	0.63	0.35	0	2.31	1.83	6.12
Na	23	2283.2	1427.61	1931.15	301.84	1581.31	8673.71	4.43	20.55
Nd	23	0	0	0	0	0	0.01	4.8	23
Ni	23	0.1	0.31	0	0	0	1.36	3.74	14.32
P	23	3986.52	1719.9	3452.55	409.78	2037.62	10939.54	3.25	12.72
Pb	23	0.13	0.29	0	0	0	1.11	2.67	6.79
Pr	23	0	0	0	0	0	0	NaN	NaN
Rb	23	1.59	0.61	1.51	0.42	0.63	3.68	1.8	5.73
Sb	23	0	0	0	0	0	0	NaN	NaN
Se	23	0.19	0.12	0.16	0.03	0.1	0.67	3.4	12.35
Sm	23	0	0	0	0	0	0	NaN	NaN
Sr	23	0.25	0.44	0.12	0.15	0	2.11	3.71	15.52
Tb	23	0	0	0	0	0	0	2.2	3.63
Te	23	0	0	0	0	0	0	NaN	NaN
Tl	23	0	0	0	0	0	0	3.13	9
Tm	23	0	0	0	0	0	0.01	4.8	23
V	23	0.07	0.1	0.03	0.05	0	0.35	1.85	2.65
Yb	23	0	0	0	0	0	0	NaN	NaN
Zn	23	9.78	8.94	7.09	3.21	0	36.59	1.98	3.84

Table S2

(a) Comparative analysis of the group of smokers vs. non-smokers based on the median content of elements in brain tissues.

Element	nonsmoker_median	smoker_median	n1	n2	statistic	p
Al	0	0	184	104	10191	0.115
As	0	0	184	104	9291.5	0.356

Ba	0	0	184	104	9840.5	0.159
Ca	46.292	43.174	184	104	10437	0.199
Cd	0.007	0.012	184	104	8605	0.145
Ce	0	0	184	104	9981.5	0.29
Co	0	0	184	104	9594	0.879
Cr	0.023	0.018	184	104	10270	0.297
Cs	0	0	184	104	10230	0.248
**Cu**	**2.485**	**1.984**	**184**	**104**	**10906**	**0.047**
Dy	0	0	184	104	9682	0.46
Er	0	0	184	104	9620	0.457
Eu	0	0	184	104	9620	0.457
Fe	39.418	47.972	184	104	8629	0.167
Gd	0	0	184	104	9748	0.38
K	2674.924	2619.631	184	104	9984	0.541
La	0	0	184	104	9394	0.259
Mg	116.746	116.712	184	104	9440	0.851
Mn	0.632	0.643	184	104	9491.5	0.911
Na	1762.815	2065.32	184	104	6127	0
Nd	0	0	184	104	9486.5	0.757
Ni	0	0	184	104	10270.5	0.25
P	2579.289	2710.052	184	104	8842	0.285
Pb	0.03	0	184	104	11233.5	0.009
Pr	0	0	184	104	9590	0.898
Rb	2.044	1.893	184	104	10319.5	0.269
Sb	0	0	184	104	9620	0.457
Se	0.199	0.188	184	104	10379	0.233
Sr	0.067	0.059	184	104	10557.5	0.141
Tb	0	0	184	104	9900.5	0.488
Tl	0	0	184	104	10069.5	0.251
Tm	0	0	184	104	9182	0.14
V	0.021	0.021	184	104	9471	0.884
Zn	9.559	8.702	184	104	10189.5	0.36

(b) Comparative analysis of medians performed using the Mann-Whitney test with Bonferroni correction in area A.

Element	nonsmoker_median	smoker_median	n1	n2	statistic	p
As	0	0	18	10	81	0.205
Ba	0	0	18	10	95	0.502
**Ca**	**74.979**	**46.372**	**18**	**10**	**130**	**0.057**
Cd	0.011	0.013	18	10	84	0.791

Ce	0	0	18	10	80	0.454
Cr	0.031	0.022	18	10	101.5	0.596
Cs	0	0	18	10	81	0.205
Cu	2.731	1.341	18	10	112	0.299
Fe	31.939	31.684	18	10	79	0.621
Gd	0	0	18	10	95	0.502
K	2301.696	2299.864	18	10	86	0.869
La	0	0	18	10	85.5	0.668
Mg	105.355	107.128	18	10	79	0.621
Mn	0.422	0.342	18	10	100	0.654
**Na**	**1989.691**	**2216.45**	**18**	**10**	**52**	**0.072**
Ni	0	0	18	10	82.5	0.64
P	2216.13	2372.309	18	10	63	0.208
Pb	0	0	18	10	103	0.496
Rb	1.417	1.682	18	10	77	0.555
Se	0.187	0.181	18	10	86	0.869
Sr	0.086	0.026	18	10	121.5	0.131
Tb	0	0	18	10	100	0.308
Tl	0	0	18	10	100	0.308
Tm	0	0	18	10	81	0.205
V	0.017	0.05	18	10	58	0.119
Zn	9.353	9.318	18	10	96	0.796

(c) Comparative analysis of medians performed using the Mann-Whitney test with Bonferroni correction for area B.

Element	nonsmoker_median	smoker_median	n1	n2	statistic	p
Al	0	0	16	10	90	0.279
**As**	**0**	**0**	**16**	**10**	**64**	**0.077**
Ba	0	0	16	10	85	0.477
Ca	28.693	33.778	16	10	80	1
**Cd**	**0**	**0.018**	**16**	**10**	**46.5**	**0.058**
Co	0	0	16	10	85	0.477
Cr	0.035	0.027	16	10	91	0.576
Cs	0	0	16	10	77	0.848
Cu	1.186	0.451	16	10	88.5	0.657
Fe	52.477	53.446	16	10	67	0.517
K	2279.781	2399.469	16	10	63	0.391
Mg	102.851	111.028	16	10	50	0.121
**Mn**	**0.431**	**0.549**	**16**	**10**	**45**	**0.068**

Na	2095.932	2360.901	16	10	57	0.241
Ni	0	0	16	10	93.5	0.434
P	2367.796	2552.548	16	10	63	0.391
Pb	0	0	16	10	84	0.833
Rb	1.573	1.486	16	10	70	0.623
Se	0.202	0.185	16	10	88	0.698
Sr	0.06	0.015	16	10	97	0.371
Tb	0	0	16	10	80.5	1
Tl	0	0	16	10	76	0.821
V	0.016	0.024	16	10	74	0.767
Zn	6.917	8.461	16	10	50	0.121

(d) Comparative analysis of medians in area C performed using the Mann-Whitney test with Bonferroni correction.

Element	nonsmoker_median	smoker_median	n1	n2	statistic	p
Al	0	0	17	10	81	0.699
As	0	0	17	10	76.5	0.22
Ca	53.973	42.416	17	10	92	0.744
Cd	0.005	0.013	17	10	67	0.363
Ce	0	0	17	10	90	0.715
**Cr**	**0.042**	**0**	**17**	**10**	**136**	**0.01**
Cs	0	0	17	10	100	0.317
Cu	3.075	2.404	17	10	101.5	0.416
Dy	0	0	17	10	90	0.49
Fe	40.977	49.053	17	10	62	0.264
Gd	0	0	17	10	76.5	0.22
K	2214.757	2300.502	17	10	74	0.604
La	0	0	17	10	76.5	0.22
Mg	102.838	103.334	17	10	80	0.824
Mn	0.481	0.455	17	10	99	0.505
Na	2228.94	2221.71	17	10	82	0.902
Nd	0	0	17	10	76.5	0.22
Ni	0	0	17	10	105	0.259
P	2440	2612.347	17	10	73	0.57
Pb	0.033	0	17	10	114	0.116
Pr	0	0	17	10	76.5	0.22
Rb	1.518	1.534	17	10	85	1
Sb	0	0	17	10	90	0.49
Se	0.174	0.215	17	10	67	0.386
Sr	0.076	0.085	17	10	88.5	0.879

Tb	0	0	17	10	73.5	0.448
Tl	0	0	17	10	110	0.069
Tm	0	0	17	10	95	0.294
V	0.024	0	17	10	106	0.267
Zn	8.686	7.321	17	10	104	0.359

(e) Comparative analysis of the medians of elements detected in region D performed using the Mann-Whitney test with Bonferroni correction.

Element	nonsmoker_median	smoker_median	n1	n2	statistic	p
Al	0	0	17	9	77	1
As	0	0	17	9	68	0.196
Ba	0	0	17	9	73	0.726
Ca	50.646	43.792	17	9	79	0.914
Cd	0	0.008	17	9	52	0.167
Ce	0	0	17	9	72	0.641
Cr	0.03	0.022	17	9	97	0.278
Cs	0	0	17	9	68.5	0.645
Cu	2.253	2.949	17	9	71.5	0.806
Fe	27.927	33.109	17	9	55	0.263
Gd	0	0	17	9	81	0.518
K	3064.621	2798.324	17	9	92	0.426
Mg	114.249	114.092	17	9	89	0.525
**Mn**	**0.432**	**0.531**	**17**	**9**	**41**	**0.058**
Na	1717.275	1856.551	17	9	50	0.164
Ni	0	0	17	9	86	0.535
P	2372.236	2331.171	17	9	76	1
Pb	0	0.031	17	9	67	0.59
Pr	0	0	17	9	81	0.518
Rb	2.29	1.742	17	9	91	0.458
Se	0.203	0.181	17	9	87	0.597
Sr	0.065	0.071	17	9	84	0.705
Tb	0	0	17	9	81	0.783
Tl	0	0	17	9	64.5	0.448
Tm	0	0	17	9	59.5	0.054
V	0.02	0.018	17	9	83.5	0.722
Zn	10.278	10.253	17	9	89	0.525

(f) Comparative analysis of medians performed using the Mann-Whitney test with Bonferroni correction in the E region.

Element	nonsmoker_median	smoker_median	n1	n2	statistic	p

Al	0	0	17	8	84	0.157
Ba	0	0	17	8	72	0.548
Ca	40.454	22.2	17	8	88	0.251
Cd	0.007	0.01	17	8	65.5	0.905
Ce	0	0	17	8	84	0.157
Cr	0.014	0.022	17	8	56	0.502
Cs	0.003	0	17	8	76	0.637
Cu	1.677	2.276	17	8	67	0.977
Dy	0	0	17	8	72	0.548
Eu	0	0	17	8	72	0.548
Fe	28.396	27.832	17	8	82	0.44
Gd	0	0	17	8	72	0.548
K	2589.465	2344.643	17	8	89	0.238
**Mg**	**114.053**	**100.91**	**17**	**8**	**106**	**0.027**
Mn	0.528	0.533	17	8	74	0.754
Na	1589.936	1688.653	17	8	49	0.288
Nd	0	0	17	8	76	0.353
Ni	0	0	17	8	77.5	0.564
P	2437.998	2273.335	17	8	83	0.406
Pb	0.063	0	17	8	97	0.083
Rb	2.031	1.741	17	8	94	0.14
Se	0.172	0.151	17	8	90	0.21
Sr	0.044	0.023	17	8	87	0.27
Tb	0	0	17	8	76	0.353
Tl	0	0	17	8	63	0.578
Tm	0	0	17	8	63	0.578
V	0.024	0.015	17	8	68	1
Zn	10.909	10.356	17	8	83	0.406

(g) Comparative analysis of medians performed using the Mann-Whitney test with Bonferroni correction in the F region.

Element	nonsmoker_median	smoker_median	n1	n2	statistic	p
Al	0	0	17	9	74	0.876
As	0	0	17	9	64	0.244
Ca	57.71	61.121	17	9	62	0.45
Cd	0.013	0.013	17	9	88	0.545
Ce	0	0	17	9	55	0.072
Cr	0.021	0.014	17	9	88.5	0.529

Cs	0.003	0	17	9	89	0.489
Cu	4.527	3.129	17	9	87	0.59
Fe	100.104	113.115	17	9	78	0.958
Gd	0	0	17	9	72	0.641
K	2800.487	2529.398	17	9	96	0.312
Mg	123.893	119.229	17	9	85	0.672
Mn	1.145	1.011	17	9	107	0.107
Na	1738.828	2227.678	17	9	49	0.148
Nd	0	0	17	9	57	0.103
Ni	0	0	17	9	87	0.549
P	2721.398	2696.184	17	9	90	0.491
Pb	0.079	0.086	17	9	71	0.781
Pr	0	0	17	9	68	0.196
Rb	2.23	1.848	17	9	91	0.458
Se	0.241	0.2	17	9	94	0.367
Sr	0.075	0.07	17	9	73.5	0.893
Tb	0	0	17	9	85.5	0.322
Tl	0	0	17	9	94.5	0.133
Tm	0	0	17	9	59.5	0.054
V	0.015	0	17	9	80.5	0.843
Zn	10.91	8.575	17	9	91	0.458

(h) Comparative analysis of medians performed using the Mann-Whitney test with Bonferroni correction in the G region.

Element	nonsmoker_median	smoker_median	n1	n2	statistic	p
Al	0	0	17	10	91	0.655
As	0	0	17	10	100	0.182
Ba	0	0	17	10	95	0.294
Ca	50.07	54.059	17	10	74.5	0.614
Cd	0.007	0.008	17	10	90	0.815
Ce	0	0	17	10	81	0.699
Cr	0.026	0.032	17	10	82	0.9
Cs	0.004	0	17	10	106.5	0.247
Cu	4.214	1.756	17	10	115	0.138
Dy	0	0	17	10	76.5	0.22
Fe	38.323	49.285	17	10	55	0.141
Gd	0	0	17	10	90	0.49
K	2816.765	2802.408	17	10	85	1
Mg	134.942	143.054	17	10	63	0.286
Mn	0.88	1.078	17	10	71	0.505

**Na**	**1446.051**	**1588.444**	**17**	**10**	**49**	**0.074**
Nd	0	0	17	10	76.5	0.22
Ni	0	0.016	17	10	63	0.245
P	4297.577	4405.07	17	10	73	0.57
Pb	0.088	0	17	10	112	0.172
Rb	2.239	2.073	17	10	88	0.902
Se	0.172	0.153	17	10	97	0.57
Sr	0.114	0.077	17	10	90	0.818
Tb	0	0	17	10	93	0.625
Tl	0	0	17	10	84.5	1
Tm	0	0	17	10	90	0.49
V	0.031	0.025	17	10	96.5	0.579
Zn	8.142	7.601	17	10	86	0.98

(i) Comparative analysis of medians was performed using the Mann-Whitney test with Bonferroni correction in the H region.

Element	nonsmoker_median	smoker_median	n1	n2	statistic	p
Al	0	0	15	9	70	0.836
As	0	0	15	9	69	0.917
Ba	0	0	15	9	72	0.491
Ca	37.751	38.153	15	9	72	0.811
Cd	0.012	0.012	15	9	75	0.668
Ce	0	0	15	9	76.5	0.291
Co	0	0	15	9	72	0.491
Cr	0.022	0.015	15	9	60.5	0.687
Cs	0	0	15	9	64	0.831
Cu	2.182	1.669	15	9	75	0.674
Dy	0	0	15	9	76.5	0.291
Er	0	0	15	9	72	0.491
Fe	38.328	42.366	15	9	64	0.861
K	2959.636	2896.315	15	9	80	0.482
La	0	0	15	9	72	0.491
Mg	145.995	138.287	15	9	79	0.519
Mn	0.817	0.731	15	9	82	0.411
Na	1411.739	1597.932	15	9	25	0.01
Nd	0	0	15	9	72	0.491
Ni	0	0	15	9	67	1
P	4381.034	4281.668	15	9	82	0.411
Pb	0.074	0	15	9	81.5	0.403
Rb	2.393	1.923	15	9	81	0.446

Se	0.179	0.133	15	9	94	0.123
Sr	0.088	0.111	15	9	71	0.858
Tb	0	0	15	9	65	0.867
Tl	0	0	15	9	76.5	0.291
V	0.028	0	15	9	75.5	0.643
Zn	6.663	6.8	15	9	72	0.815

(j) Comparative analysis of the content of elements in brain area I for the studied populations (smoker/non-smoker) in area I.

Element	nonsmoker_median	smoker_median	n1	n2	statistic	p
Al	0	0	18	10	81	0.503
As	0	0	18	10	90	1
Ca	45.587	50.741	18	10	77.5	0.565
Cd	0.009	0.029	18	10	67	0.277
Ce	0	0	18	10	115	0.079
Co	0	0	18	10	81	0.205
Cr	0.013	0.015	18	10	85	0.827
Cs	0	0	18	10	101	0.568
Cu	2.319	1.172	18	10	107	0.427
Fe	56.677	65.051	18	10	75	0.494
Gd	0	0	18	10	95	0.502
K	2522.684	2472.463	18	10	88	0.944
Mg	119.417	128.702	18	10	72	0.408
Mn	0.776	0.763	18	10	92	0.944
Na	1765.695	2044.602	18	10	29	0.003
Nd	0	0	18	10	100	0.308
Ni	0.007	0.003	18	10	90	1
P	2866.919	2753.569	18	10	103	0.555
Pb	0.038	0	18	10	129.5	0.051
Rb	2.202	2.102	18	10	92	0.944
Se	0.21	0.234	18	10	91	0.981
Sr	0.067	0.074	18	10	75.5	0.501
Tb	0	0	18	10	109	0.244
Tl	0	0	18	10	105	0.196
Tm	0	0	18	10	94.5	0.753
V	0.018	0.012	18	10	85	0.824
Zn	9.266	11.501	18	10	64	0.226

(k) Comparative analysis of the content of elements in tissue J for the studied populations (smoker/non-smoker).

Element	nonsmoker_median	smoker_median	n1	n2	statistic	p
Al	0	0	14	9	65	0.872
As	0	0	14	9	67.5	0.476
Ca	42.919	19.755	14	9	81	0.264
Cd	0.004	0.023	14	9	53.5	0.554
Ce	0	0	14	9	58	0.668
Co	0	0	14	9	56	0.247
Cr	0.023	0.034	14	9	53	0.544
Cs	0	0	14	9	67	0.775
Cu	3.311	0	14	9	76.5	0.403
Fe	113.031	126.39	14	9	56	0.688
K	3269.317	3514.111	14	9	64	0.975
Mg	129.494	146.496	14	9	53	0.557
Mn	1.071	1.105	14	9	58	0.781
**Na**	**1442.755**	**2402.024**	**14**	**9**	**20**	**0.005**
Nd	0	0	14	9	56	0.247
Ni	0.002	0	14	9	76.5	0.352
P	2993.915	3144.286	14	9	46	0.305
Pb	0	0	14	9	63	1
Rb	2.662	2.275	14	9	75	0.477
Se	0.252	0.289	14	9	64	0.975
Sr	0.032	0.028	14	9	58.5	0.797
Tb	0	0	14	9	65	0.872
Tl	0	0	14	9	60.5	0.861
V	0.032	0.033	14	9	65	0.922
Zn	9.087	9.477	14	9	55	0.643

(l) Comparative analysis of the content of elements in K tissue for the studied populations (smoker/non-smoker).

Element	nonsmoker_median	smoker_median	n1	n2	statistic	p
Al	0	0	18	10	125	0.03
As	0	0	18	10	100	0.308
Ca	46.753	37.674	18	10	110	0.35
Cd	0.008	0.01	18	10	87	0.902
Ce	0	0	18	10	115	0.079
Co	0	0	18	10	100	0.308
Cr	0.015	0.005	18	10	114.5	0.235

Cs	0.004	0	18	10	97	0.739
Cu	2.423	3.242	18	10	86.5	0.885
Fe	36.315	32.847	18	10	106	0.464
Gd	0	0	18	10	95	0.502
K	2870.863	3079.806	18	10	66	0.265
La	0	0	18	10	81	0.205
Mg	108.678	116.648	18	10	69	0.332
Mn	0.513	0.429	18	10	117	0.208
Na	1770.865	2086.348	18	10	50	0.057
Nd	0	0	18	10	105	0.196
Ni	0.005	0.005	18	10	100	0.638
P	2356.169	2524.289	18	10	70	0.356
Pb	0.033	0.063	18	10	81.5	0.695
Pr	0	0	18	10	105	0.196
Rb	2.22	2.142	18	10	83	0.759
Se	0.206	0.208	18	10	91	0.981
Sr	0.087	0.038	18	10	115.5	0.228
Tb	0	0	18	10	72	0.327
Tl	0	0	18	10	100	0.308
Tm	0	0	18	10	81	0.205
V	0.02	0.024	18	10	75	0.48
Zn	10.925	11.253	18	10	98	0.724

Table S2 (m) Comparative analysis of the content of elements in spinal cord tissue for the studied populations (smoker/non-smoker).

Element	nonsmoker_median	smoker_median	n1	n2	statistic	p
Al	0	0	15	8	53.5	0.635
As	0	0	15	8	64	0.523
Ba	0	0	15	8	52.5	0.201
Ca	50.4056	64.6309	15	8	54	0.721
Cd	0.0063	0.03965	15	8	37	0.134
Ce	0	0	15	8	60.5	1
Cr	0.0363	0.09645	15	8	34	0.101
Cs	0	0	15	8	65	0.66
Cu	0	0.07925	15	8	57	0.862
Fe	13.9323	19.78975	15	8	43	0.294
K	1965.259	1987.355	15	8	44	0.325
Lu	0	0	15	8	52.5	0.201
Mg	117.0297	135.3224	15	8	34	0.101
Mn	0.6008	0.7024	15	8	53	0.675

Na	1830.975	2200.62	15	8	28	0.04
Nd	0	0	15	8	64	0.523
Ni	0	0	15	8	65	0.748
P	3319.564	3964.707	15	8	30	0.056
Pb	0	0	15	8	66	0.687
Rb	1.5506	1.4039	15	8	61	0.975
Se	0.1576	0.1771	15	8	33	0.087
Sr	0.1175	0.11885	15	8	56	0.82
Tb	0	0	15	8	65	0.66
Tl	0	0	15	8	59.5	1
Tm	0	0	15	8	64	0.523
V	0.0315	0.0243	15	8	58	0.922
Zn	6.5622	9.10775	15	8	31	0.065

Table S3. Detailed characteristic of the ICP MS method.

**Sample analysis**	
ICP MS spectrometer	PlasmaQuant MS Q (Analytik Jena, Germany)
radio frequency (RF) power [kW]	1.20
argon gas flows [L min^{-1}]	9.0 (plasma), 1.5 (auxiliary), 1.02 (nebulizer)
sampling depth [mm]	5.0
spray chamber type (temp.)	double pass Scott-type (+3°C)
scans/replicates	20/5
dwell time [ms]	20
polyatomic interferences correction	integrated Collision Reaction Cell (iCRC)
collision cell (helium flow)	Al, Cd, Ce, Co, Cu, Dy, Er, Eu, Gd, La, Mn, Nd, Ni, Pr, Sm, Tm, V, Zn (80 mL min^{-1}),<br>Ca, Fe, K, Mg, Na, P (150 mL min^{-1})
reaction cell (hydrogen flow)	As, Cr, Se (80 mL min^{-1}),
no gas correction	Be, Ba, Cs, Rb, Sb, Sr, Pb, Tl
**Quality control**	
internal standard	5 μg L^{-1} (^{6}Li, Sc, Y, Rh, Ir, Bi)
control of a series of analyses	blank, reagent blanks, control sample, CRMs
uncertainty (k=2)	<10%; including a sample preparation <20%
traceability	CRMs, standard addition (recovery 80-120%)
certified standard materials	Table S2
method detection limits<br>(min–max) [μg g^{-1}] *	Al (0.46–1.4); As (0.009–0.028); Ba (0.054–0.160);<br>Be (0.003–0.009); Ca (2.8–8.4); Cd (0.003–0.009);<br>Ce (0.001–0.004); Co (0.002–0.005); Cr (0.005–0.014);<br>Cs (0.003–0.008); Cu (0.036–0.107); Dy (0.001–0.002);<br>Er (0.001–0.003); Eu (0.001–0.002); Fe (0.70–2.1);<br>Gd (0.002–0.004); K (2.9–8.8); La (0.004–0.011);<br>Mg (1.6–4.7); Mn (0.020–0.061); Na (1.7–5.1);<br>Nd (0.002–0.005); Ni (0.001–0.004); P (3.6–11);<br>Pb (0.005–0.015); Pr (0.001–0.002); Rb (0.009–0.026);

	Sb (0.013–0.040); Se (0.019–0.056); Sm (0.002–0.004); Sr (0.007–0.021); Tb (0.0003–0.001); Tl (0.001–0.002); Tm (0.004–0.011); V (0.003–0.009); Zn (0.040–0.12);

* – min-max range of method limit detection, corresponding to max and min sample weight

Table S4. The full list of CRMs used to validate the ICP MS measurements.

Matrix	CRM
water	NIST SRM 1643f, CRM-449, CRM-582
soil	NIST SRM 2709, NIST SRM 2709a, AN-ZP01, AN-ZP02
sediment	LGC-6187, IAEA-405, BCR-667, LKSD-1-4, CNS-392
plant	NIST SRM 1515, AN-BM01, NIST SRM 1547, NIST SRM 1573a, NIST SRM 1575a, INCT-OBTL-5, INCT-PVTL-6, BCR-402,
wood	NIST SRM 2791, NIST SRM 2790, IPE-240
mushroom	CS-M-3, IPE 120,
tissue	BCR-668, DB001, BCR-185R, ERM-BB184, Seronorm L1, DA-120a, BCR-627,
food, feed	NIST SRM 1568b, BCR-129, ERM-CD281, AN-BM02, LGC-7162, NIST SRM 1570a, ERM-BD150, INCT-TL-1, INCT-CF-3, INCT-SBF-4
materials	NIST SRM 610, NIST SRM 612, NIST SRM 981

Table S5. DL values for chemical elements measured by ICP-MS.

Pierwiastek chemiczny	IDL [ng/g]	Pierwiastek chemiczny	IDL [ng/g]
Al	0.79	Lu	0.0082
V	0.0051	Na	2.9
Mn	0.035	Mg	2.7
Co	0.0029	P	6.2
Ni	0.0021	K	5
Cu	0.061	Ca	4.8
Zn	0.069	Fe	1.2
Cd	0.0051	Be	0.005
La	0.0065	B	0.031
Ce	0.0024	Rb	0.015
Pr	0.001	Sr	0.012
Nd	0.0031	Sb	0.023
Sm	0.0025	Te	0.0057
Eu	0.001	Cs	0.0048
Gd	0.0025	Ba	0.093
Tb	0.0005	Tl	0.001
Dy	0.001	Pb	0.0087
Ho	0.013	Cr	0.0078
Er	0.0017	As	0.016
Tm	0.0065	Se	0.032
Yb	0.0014		

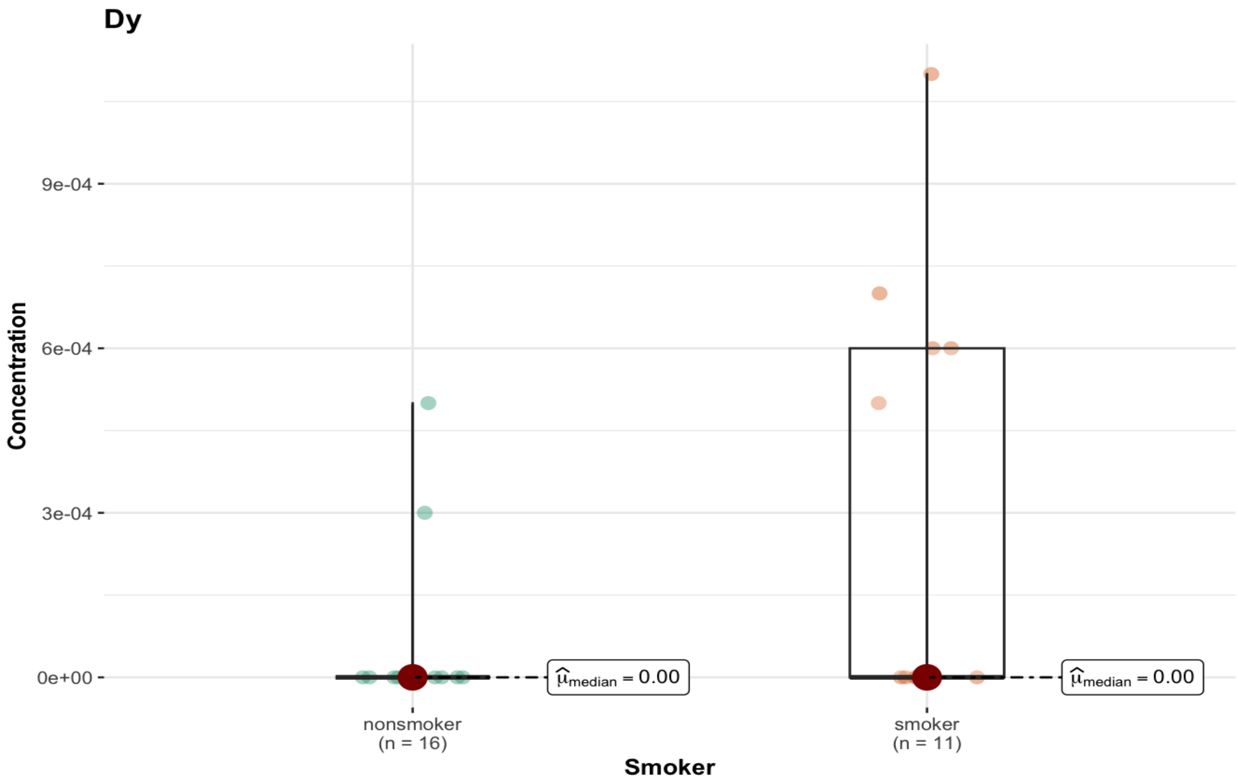

Fig. S1. Comparison of statistically significant difference in median values of Dy content in the right lung tissue of smokers (smokers) and nonsmokers (nonsmokers).

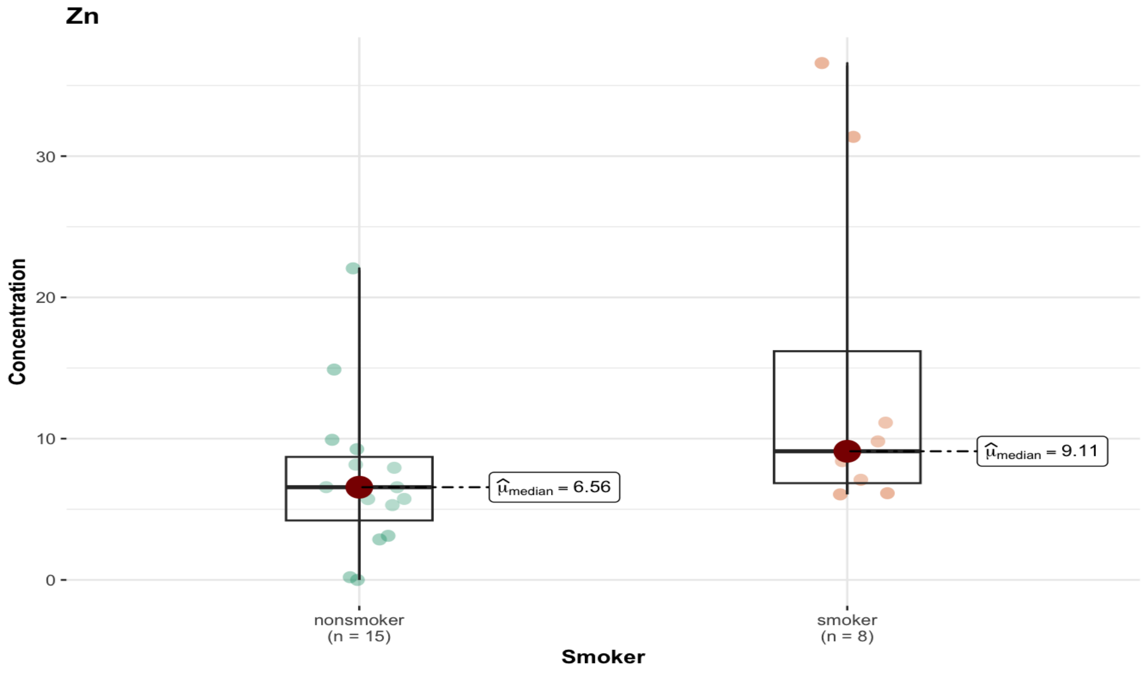

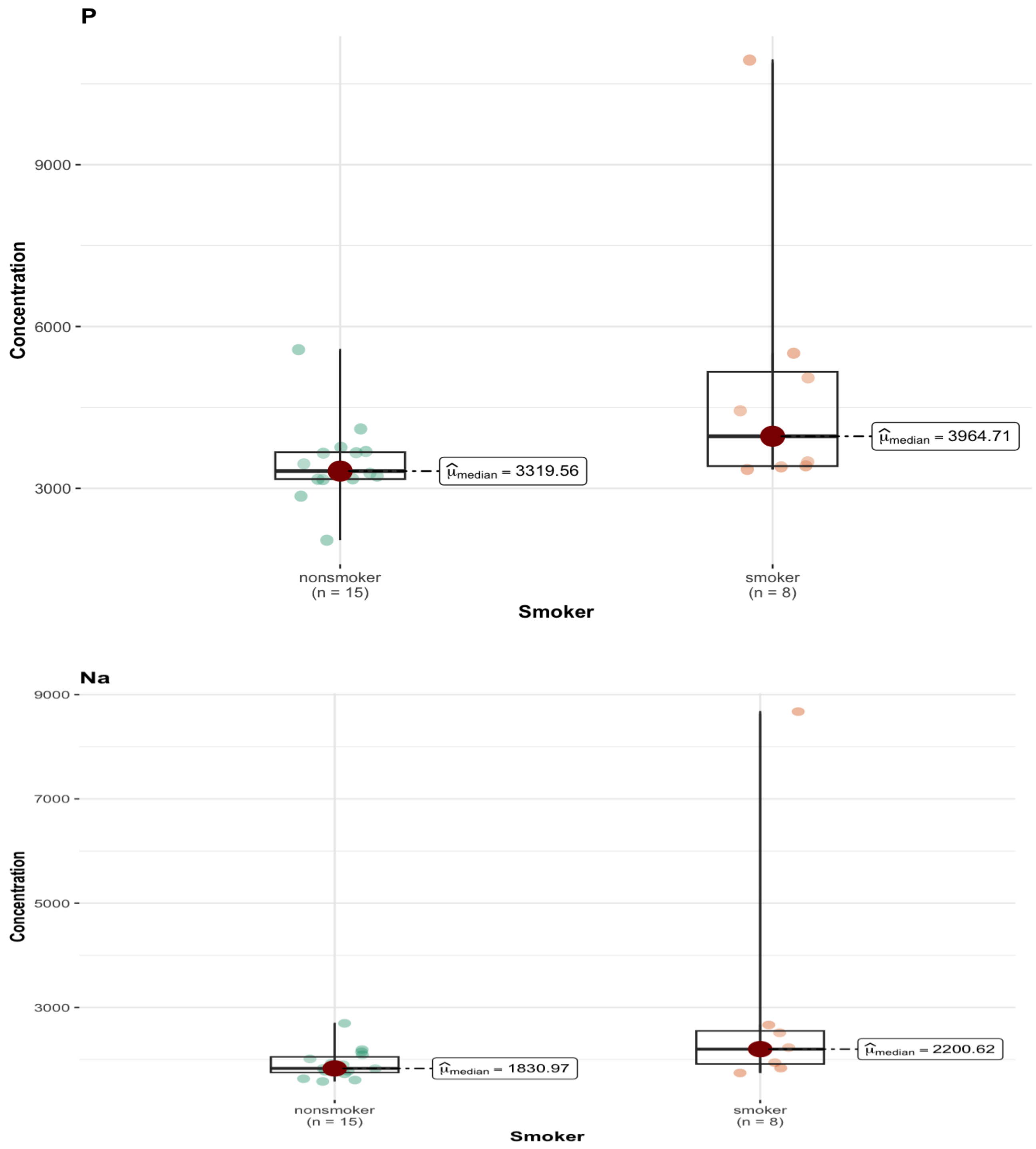

Fig. S2. Graphs comparing statistically significant difference in the median level for elements for which $p < 0.1$ in the spinal cord region.

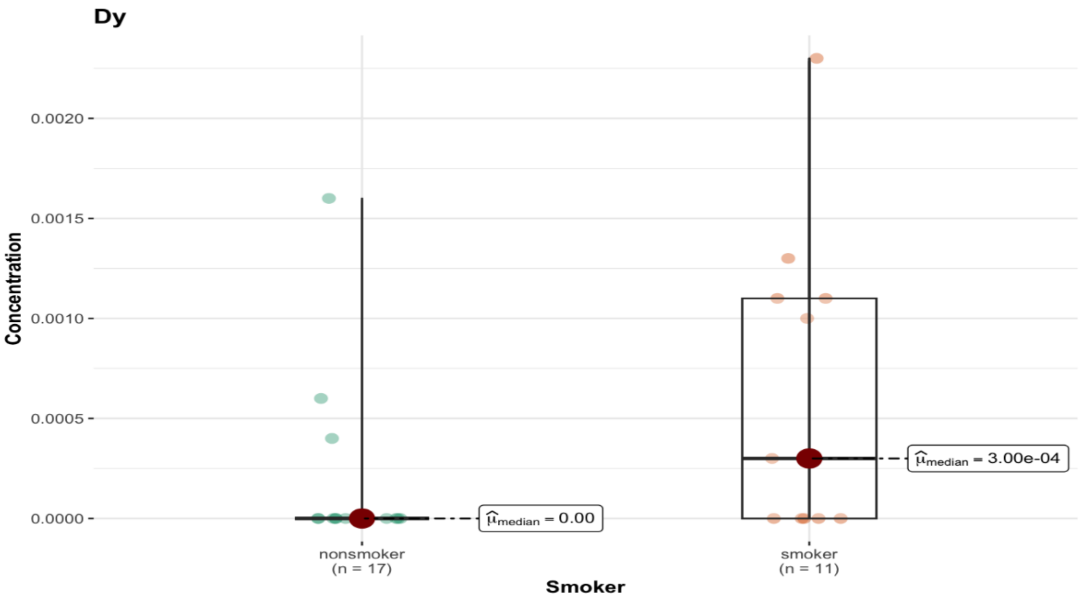

Dy
Concentration
0.0020
0.0015
0.0010
0.0005
0.0000
$\widehat{\mu}_{median} = 0.00$
$\widehat{\mu}_{median} = 3.00e-04$
nonsmoker
(n = 17)
smoker
(n = 11)
Smoker

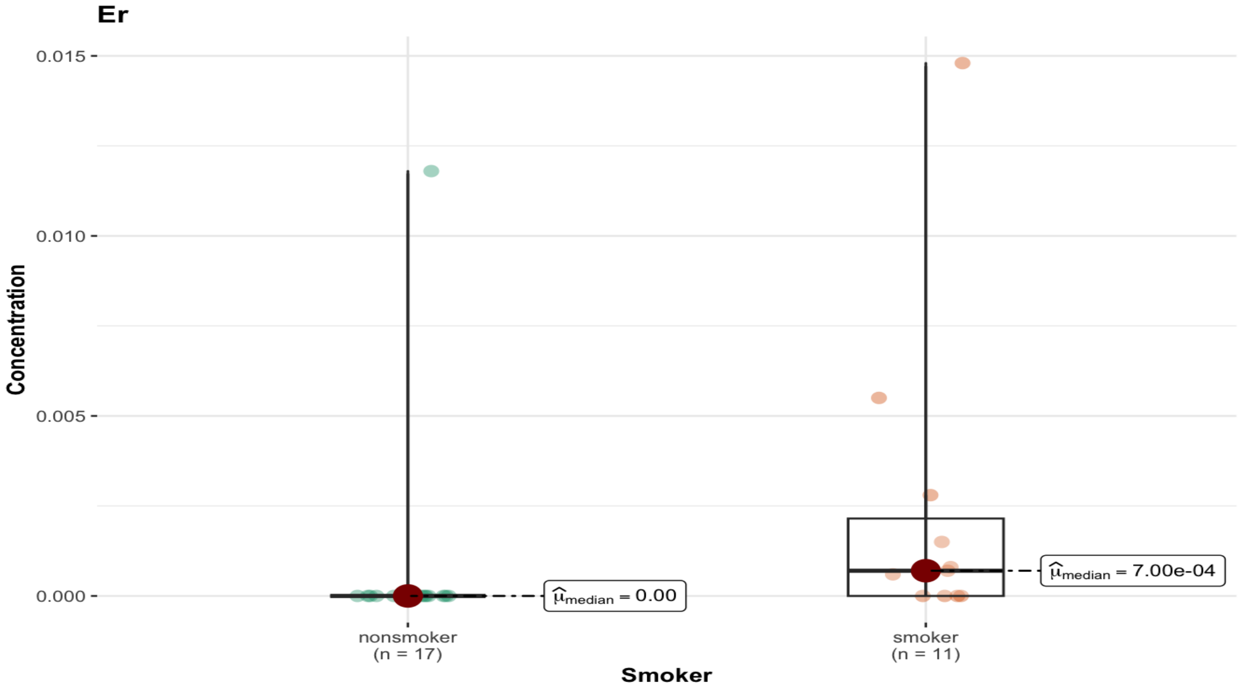

Er
Concentration
0.015
0.010
0.005
0.000
$\hat{\mu}_{median} = 0.00$
$\hat{\mu}_{median} = 7.00e-04$
nonsmoker
(n = 17)
smoker
(n = 11)
Smoker

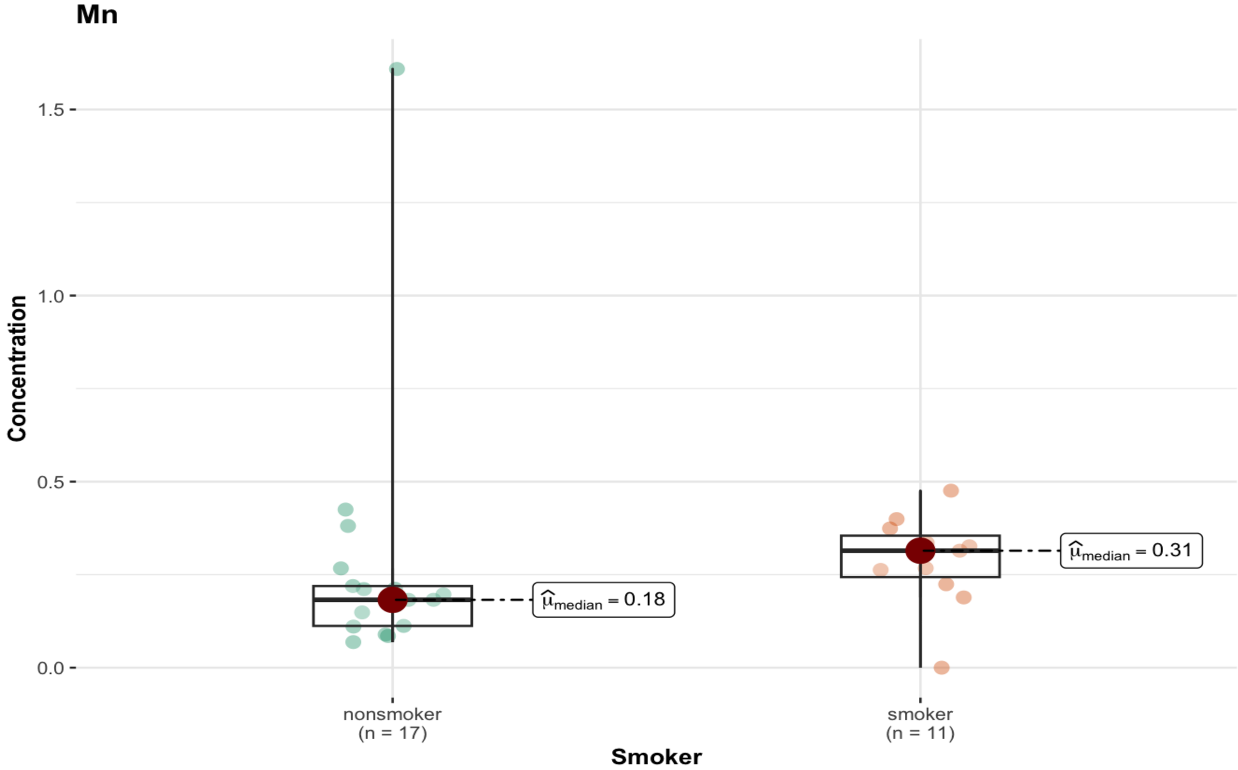

Mn
Concentration
1.5
1.0
0.5
0.0
$\hat{\mu}_{median} = 0.18$
$\hat{\mu}_{median} = 0.31$
nonsmoker
(n = 17)
smoker
(n = 11)
Smoker

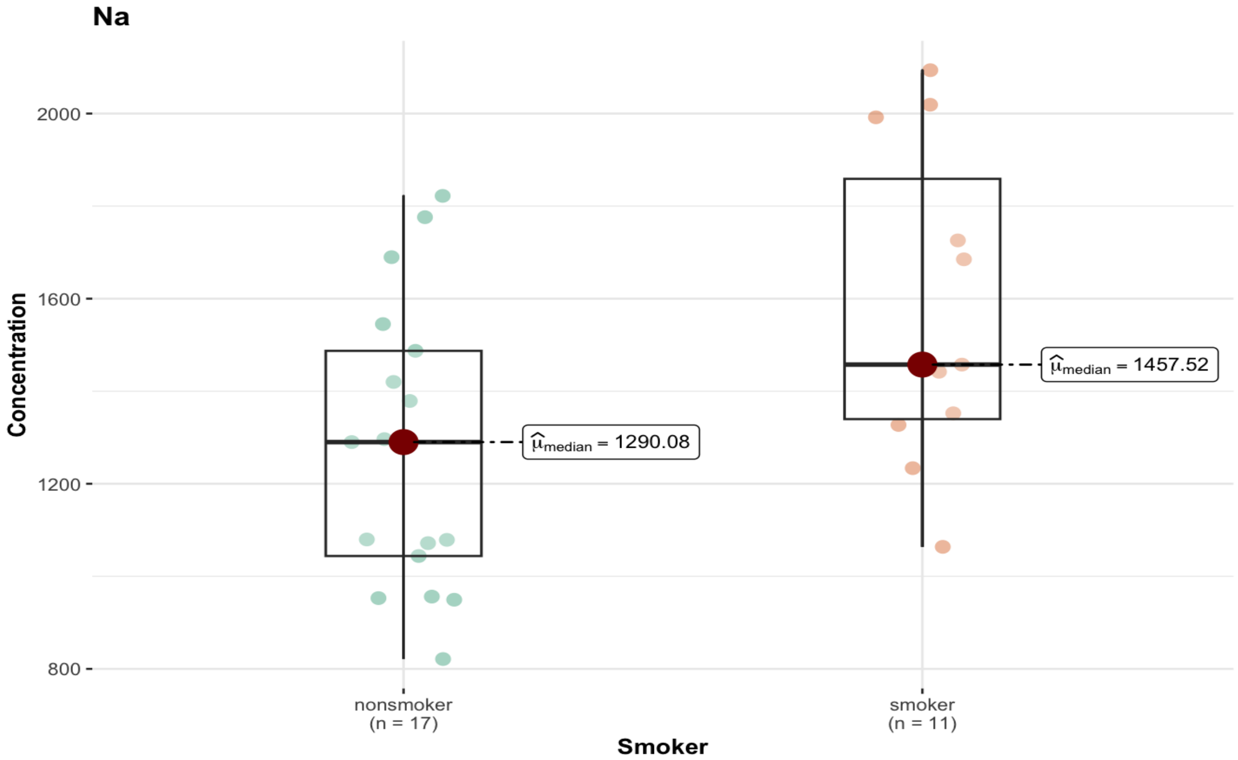

Na
Concentration
2000
1600
1200
800
$\hat{\mu}_{median}$ = 1290.08
$\hat{\mu}_{median}$ = 1457.52
nonsmoker
(n = 17)
smoker
(n = 11)
Smoker

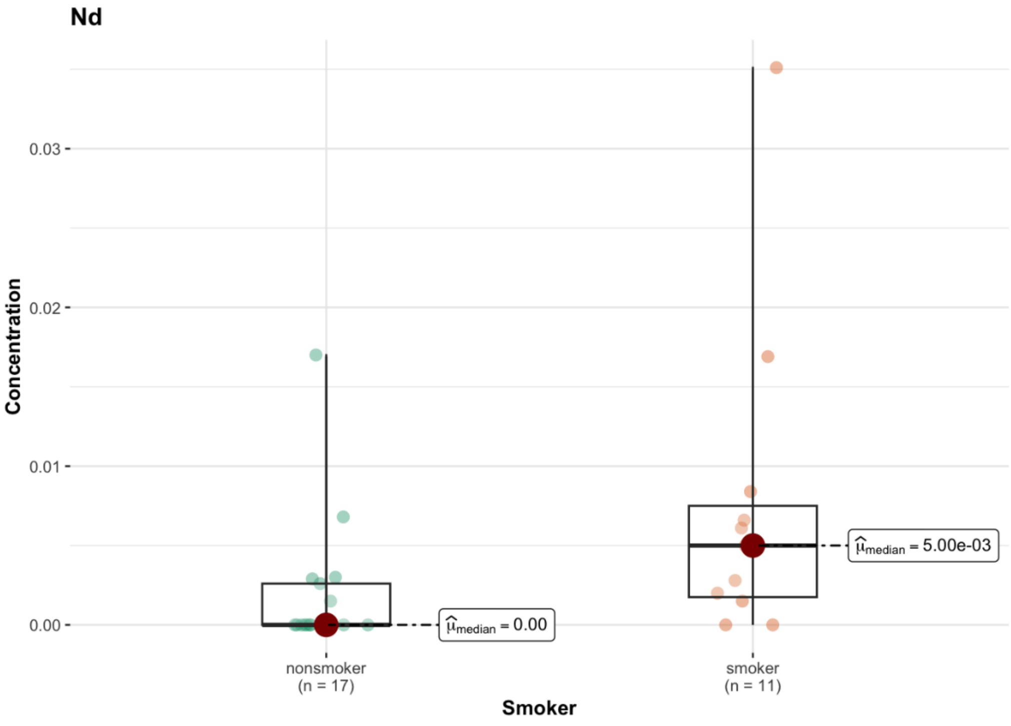

Nd
Concentration
0.03
0.02
0.01
0.00
$\hat{\mu}_{median}$ = 0.00
$\hat{\mu}_{median}$ = 5.00e-03
nonsmoker
(n = 17)
smoker
(n = 11)
Smoker

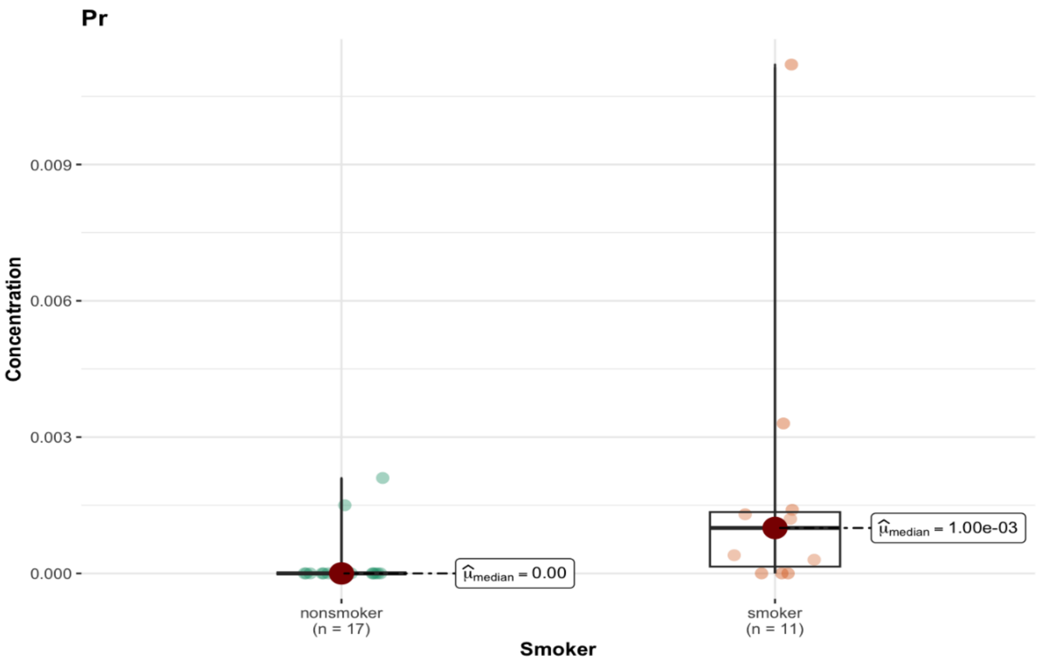
Pr
Concentration
0.009
0.006
0.003
0.000
$\widehat{\mu}_{median} = 0.00$
$\widehat{\mu}_{median} = 1.00e-03$
nonsmoker
(n = 17)
smoker
(n = 11)
Smoker

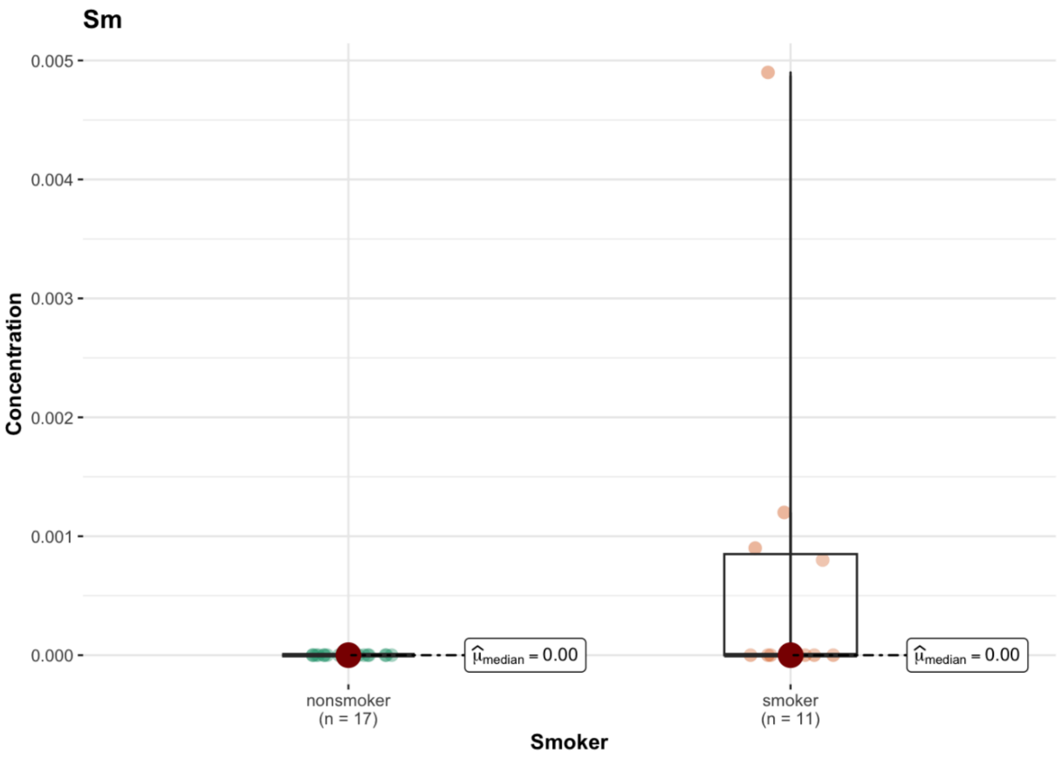
Sm
Concentration
0.005
0.004
0.003
0.002
0.001
0.000
$\widehat{\mu}_{median} = 0.00$
$\widehat{\mu}_{median} = 0.00$
nonsmoker
(n = 17)
smoker
(n = 11)
Smoker

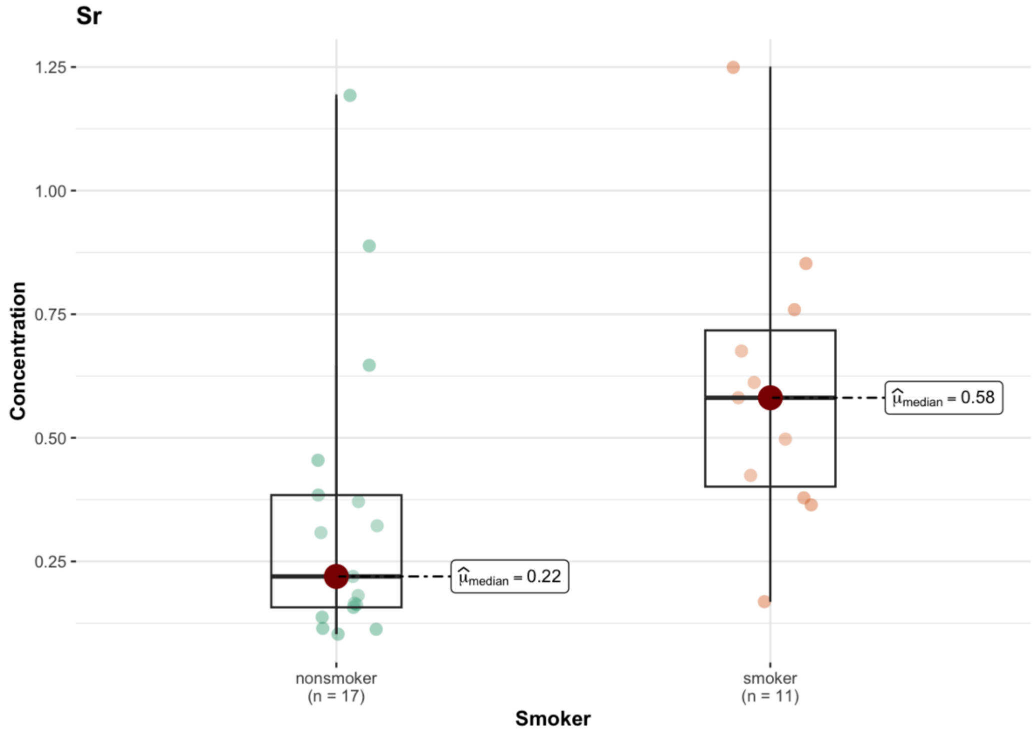

Fig. S3. Box-and-whisker plots for elements that achieved a statistically significant difference in concentration in the left lung tissue of smokers and nonsmokers.
